# Targeting invadopodia-mediated breast cancer metastasis by using ABL kinase inhibitors

**DOI:** 10.18632/oncotarget.25243

**Published:** 2018-04-24

**Authors:** Tomer Meirson, Alessandro Genna, Nikola Lukic, Tetiana Makhnii, Joel Alter, Ved P. Sharma, Yarong Wang, Abraham O. Samson, John S. Condeelis, Hava Gil-Henn

**Affiliations:** ^1^ Laboratory of Cell Migration and Invasion, The Azrieli Faculty of Medicine, Bar-Ilan University, Safed, 1311502, Israel; ^2^ Drug Discovery Laboratory, The Azrieli Faculty of Medicine, Bar-Ilan University, Safed, 1311502, Israel; ^3^ Department of Anatomy and Structural Biology, Albert Einstein College of Medicine, Bronx, New York 10461, USA; ^4^ Gruss Lipper Biophotonics Center, Albert Einstein College of Medicine, Bronx, New York 10461, USA; ^5^ Integrated Imaging Program, Albert Einstein College of Medicine, Bronx, New York 10461, USA

**Keywords:** ABL kinases, inhibitors, invadopodia, in vivo, cancer metastasis

## Abstract

Metastatic dissemination of cancer cells from the primary tumor and their spread to distant sites in the body is the leading cause of mortality in breast cancer patients. While researchers have identified treatments that shrink or slow metastatic tumors, no treatment that permanently eradicates metastasis exists at present. Here, we show that the ABL kinase inhibitors imatinib, nilotinib, and GNF-5 impede invadopodium precursor formation and cortactin-phosphorylation dependent invadopodium maturation, leading to decreased actin polymerization in invadopodia, reduced extracellular matrix degradation, and impaired matrix proteolysis-dependent invasion. Using a mouse xenograft model we demonstrate that, while primary tumor size is not affected by ABL kinase inhibitors, the *in vivo* matrix metalloproteinase (MMP) activity, tumor cell invasion, and consequent spontaneous metastasis to lungs are significantly impaired in inhibitor-treated mice. Further proteogenomic analysis of breast cancer patient databases revealed co-expression of the Abl-related gene (Arg) and cortactin across all hormone- and human epidermal growth factor receptor 2 (HER2)-receptor status tumors, which correlates synergistically with distant metastasis and poor patient prognosis. Our findings establish a prognostic value for Arg and cortactin as predictors of metastatic dissemination and suggest that therapeutic inhibition of ABL kinases may be used for blocking breast cancer metastasis.

## INTRODUCTION

While primary tumors can often be surgically removed and are usually responsible for only a small percentage of cancer deaths, complications associated with distant metastasis are the primary cause of mortality from cancer and therefore serve as an important target for potential therapeutic intervention. Metastatic cancer cells must penetrate through several barriers to escape the primary tumor and gain entry into the bloodstream in order to spread to other tissues. Invasive cancer cells penetrate these barriers by forming invadopodia, F-actin rich protrusions that localize matrix-degrading activity to cell-substrate contact points and represent sites in which cell signaling, proteolytic, adhesive, cytoskeletal, and membrane-trafficking pathways physically converge [1–3]. Invadopodia were identified in a number of invasive cancer cell lines, such as breast, head and neck, prostate, fibrosarcoma, and melanoma [4] as well as in primary tumor cells [5–7]. Recent evidence also demonstrates direct molecular links between invadopodia assembly and function and metastasis in mice models [8–10] and human patients [11].

The ABL family of non-receptor tyrosine kinases, which includes c-Abl (Abl; ABL1) and Abl-related gene (Arg; ABL2) link diverse stimuli from cell surface growth factor and adhesion receptors to signaling pathways controlling cell proliferation, survival, adhesion, migration and invasion [12–15]. Abl kinase was originally discovered as an oncogene in the Abelson murine leukemia virus (v-Abl) [16] and was later on identified as an oncogene associated with chromosomal translocation in BCR-ABL positive human leukemias [17]. Similar chromosomal translocation of the TEL transcription factor gene next to Arg have been detected in rare cases of acute myeloid leukemias [18–21]. These fusion genes encode constitutively activated forms of Abl and Arg kinases that are required for cellular transformation. Accumulating data support a role for ABL family kinases in the progression of solid tumors. Unlike leukemia, activation of ABL kinases in solid tumors is not linked to chromosomal translocation events, but is rather characterized by their enhanced expression and activation due to amplification, increased gene or protein expression, or increased activity in response to stimulation by oncogenic tyrosine kinase and chemokine receptors, oxidative stress, and metabolic stress [13].

The development of ABL tyrosine kinase inhibitors that were originated against the oncogenic BCR-ABL protein for the treatment of chronic myeloid leukemia (CML) is the most successful example of molecular targeted therapy to date. Inhibitors of ABL kinases are classified into three main classes on the basis of their mechanism of action: type 1 inhibitors target the active conformation of the kinase domain (dasatinib and bosutinib), whereas type 2 inhibitors stabilize the inactive conformation of the kinase domain, preventing its activation (imatinib, nilotinib and ponatinib). The third category of inhibitors includes allosteric inhibitors, which do not compete for ATP binding but rather bind to regulatory domains to inhibit kinase activity. Among these are GNF-2 and GNF-5, which target the myristate-binding pocket in the C-lobe of the kinase domain. In contrast to ATP-competitive inhibitors that target multiple kinases, the allosteric inhibitors are highly selective for Abl and Arg. Although ABL kinase inhibitors have been shown to decrease metastatic colonization of breast cancer cells to bone [14], colonization of melanoma cancer cells to lungs [13], and extravasation and colonization of lung cancer cells to the lung parenchyma [22], the direct effect of these inhibitors on invadopodia-mediated breast cancer invasiveness and consequent metastatic dissemination *in vivo* has never been examined.

We have previously shown that Arg localizes to invadopodia in breast cancer cells, where it controls actin polymerization, matrix degradation, and consequent tumor cell invasion. Arg regulates the maturation of invadopodia by linking activation of epidermal growth factor receptor (EGFR) and Src kinase to tyrosine phosphorylation of cortactin, which is required for Arp2/3 complex-dependent actin polymerization [23]. Stable knockdown of Arg in MDA-MB-231 breast cancer cells enhances the growth of xenograft tumors owing to increased cell proliferation. Despite having larger tumors, the Arg knockdown tumor-bearing mice exhibit significant reduction in tumor cell invasion, intravasation into blood vessels, and spontaneous metastasis to lungs [8].

Based on our previous findings, we hypothesized that Arg kinase could be used as a therapeutic candidate for inhibition of breast cancer metastasis. Here, we demonstrate that inhibition of ABL family kinases by imatinib, nilotinib, or GNF-5 blocked invadopodia formation and function and consequent *in vivo* breast cancer invasiveness. ABL kinase inhibitors significantly reduced invadopodium precursor formation as well as cortactin tyrosine phosphorylation and consequent actin polymerization, extracellular matrix degradation, and three-dimensional (3D) tumor cell invasion in invadopodia of inhibitor-treated breast cancer cells. Additionally, while primary tumor growth was not affected by ABL kinase inhibitors, *in vivo* matrix metalloproteinase (MMP) activation, tumor cell invasion, and consequent pulmonary metastasis were severely impaired in breast tumor bearing mice that were treated with ABL kinase inhibitors. Careful proteogenomic analysis of breast cancer patient databases revealed a correlation between increased Arg and cortactin expression to metastatic dissemination and poor patient prognosis. These data suggest that Arg kinase may serve as a novel prognostic and therapeutic target for breast cancer metastasis.

## RESULTS

### Mechanism of tyrosine kinase inhibition by imatinib, nilotinib, and GNF-5

**Figure 1:**
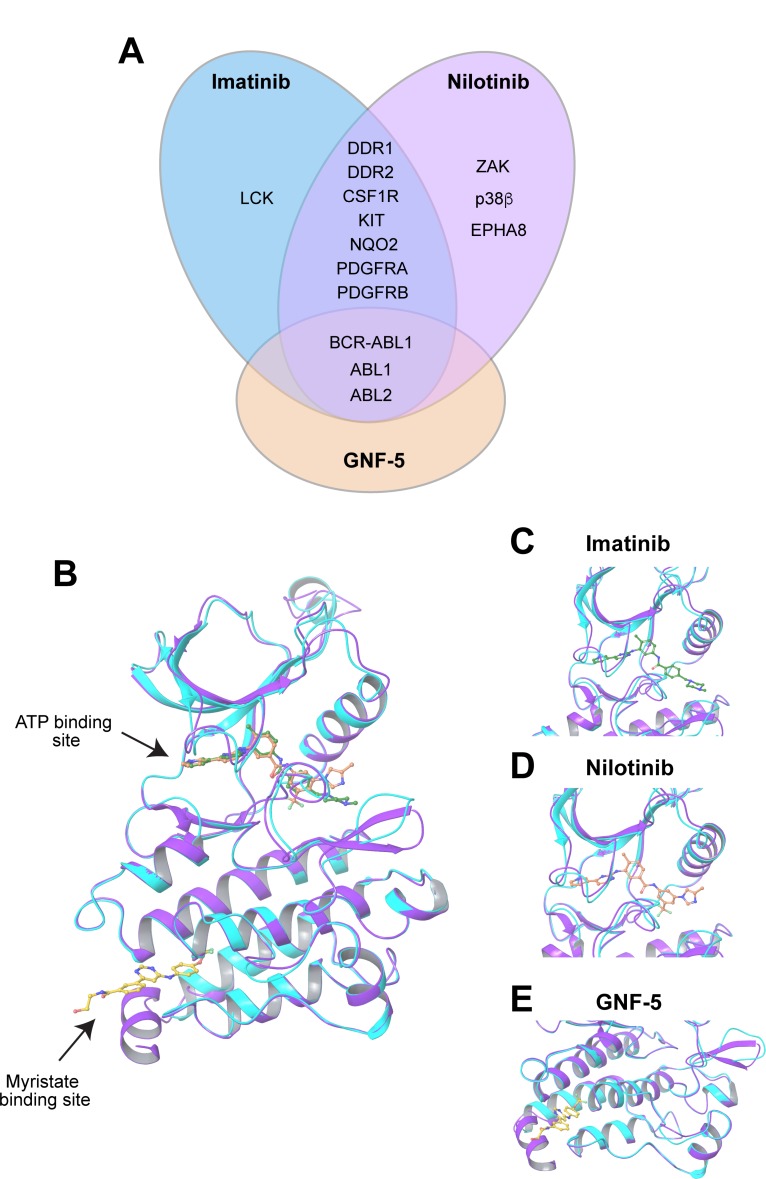
Imatinib, nilotinib, and GNF-5 inhibit the ABL family of non-receptor tyrosine kinases (**A**) Specificity of ABL kinase inhibitors used in this study: imatinib, nilotinib, and GNF-5. LCK, lymphocyte-specific kinase; DDR, discoidin domain receptor; CSF1R, colony stimulating factor 1 receptor; KIT, stem cell growth factor receptor; NQO2, NADPH dehydrogenase, quinone 2; PDGFR, platelet-derived growth factor receptor; ZAK, Sterile alpha motif and leucine zipper containing kinase AZK; p38β, mitogen activated protein kinase 11; EPHA8, ephrin receptor 8; BCR, breakpoint cluster region protein; ABL1, ABL proto oncogene 1, ABL2, ABL proto oncogene 2 [14, 15, 31]. (**B**) Graphical ribbon representation of Arg kinase domain (cyan) structurally aligned with Abl kinase domain (purple) and complexed with imatinib (green), nilotinib (orange) and GNF-5 (yellow), represented by ball-and-stick models. Imatinib and nilotinib occupy the ATP binding cleft between the N-terminal and C-terminal lobes, while GNF-5 is situated at the myristate pocket at the C-terminal lobe of the kinase domain. (**C**–**E**) Close-up images of imatinib (C), nilotinib (D), and GNF-5 (E) with the overlapped Arg/Abl kinase domains.

To evaluate whether inhibition of Arg kinase activity could potentially suppress invadopodia formation and function and consequent *in vivo* breast cancer metastasis, we chose three ABL kinase inhibitors, imatinib, nilotinib, and GNF-5. Imatinib mesylate (Gleevec, STI-571; Novartis) is an FDA approved tyrosine kinase inhibitor that was originally developed against BCR-ABL1 for the treatment of CML and Ph+ (Philadelphia positive) leukemia patients in chronic phase [24, 25]. Imatinib targets the ATP binding site within the kinase domain of BCR-ABL1 and its binding stabilizes the inactive conformation of the kinase. Nilotinib (Tasigna, AMN107; Novartis) is an FDA approved tyrosine kinase inhibitor and an ATP competitor that is approximately 20-fold more potent than imatinib, and is used as a second line therapy in patients with imatinib resistant mutations. Similarly to imatinib, nilotinib stabilizes the inactive, DFG-out conformation of the BCR-ABL1 kinase [26–28]. GNF-5 is a pre-clinical, non-ATP competitive, allosteric kinase inhibitor that binds to the myristate pocket near the C-terminus of the ABL kinase domain and transmits structural changes to the ATP binding site. As a result, GNF-5 can sensitize mutant BCR-ABL1 to inhibition by ATP-competitive inhibitors such as imatinib or nilotinib [29, 30]. While GNF-5 is highly selective for Abl, Arg, and BCR-ABL, imatinib and nilotinib show broader tyrosine kinase specificities that include, in addition to Arg and Abl, kinases such as PDGFRA and PDGFRB, CSF1R, c-KIT, and others [14, 15, 31] (Figure 1A).

Abl and Arg share 93% amino acid sequence identity within their kinase domains [32]. While several structural studies characterized the binding of Abl to imatinib and nilotinib and of Arg to imatinib [28, 30, 32], no crystal structure of Abl with GNF-5 or Arg with nilotinib or GNF-5 exists at present. To evaluate the structural homology between the two kinases and the molecular structure by which they bind to each of the inhibitors, we performed structural alignment of the kinase domains of Abl and Arg including the three inhibitors. Alignment of the kinase domains of the two kinases showed high structural homology with a root-mean-square deviation (RMSD) of 0.92 Å based on the Cα atoms (Figure 1B–1E). Together, these observations suggest that the ABL kinase inhibitors imatinib, nilotinib, and GNF-5 could bind and inhibit Arg with specificity and selectivity that are similar to their effect towards Abl kinase.

### Tks5 and cortactin co-localize to invadopodium precursors and mature invadopodia and can be used as their markers

Invadopodia formation and function have now been linked to *in vivo* breast cancer invasiveness [8–10]. To examine the effect of ABL kinase inhibitors on invadopodial functions *in vitro* and on consequent invadopodia-mediated breast cancer metastasis in mice, we used the MDA-MB-231 cell line, a triple-negative basal-like human breast adenocarcinoma cell line that forms functional invadopodia in culture and is also widely used for studying metastasis *in vivo* because of its ability to grow orthotopic tumors that spontaneously metastasize to the lungs in mice.

Cortactin and Tks5 are frequently used as markers of invadopodium precursors, and co-localization of these two proteins with degraded matrix (gelatin) is used as a marker for mature, active invadopodia [23, 33–43]. MDA-MB-231 cells were plated on fluorescently labeled gelatin matrix and labeled for Tks5 and cortactin as invadopodium precursor markers. X-Z confocal imaging clearly showed that cortactin and Tks5 co-localize to invadopodium precursors as well as to matrix degrading invadopodia at the ventral side of the cell facing the matrix (Supplementary Figure 1). These data suggest that co-localization of cortactin and Tks5, or cortactin, Tks5, and degraded gelatin, can be used as a method for quantification of precursors or mature invadopodia, respectively.

### Invadopodium precursor formation and maturation are compromised by ABL kinase inhibitors

**Figure 2:**
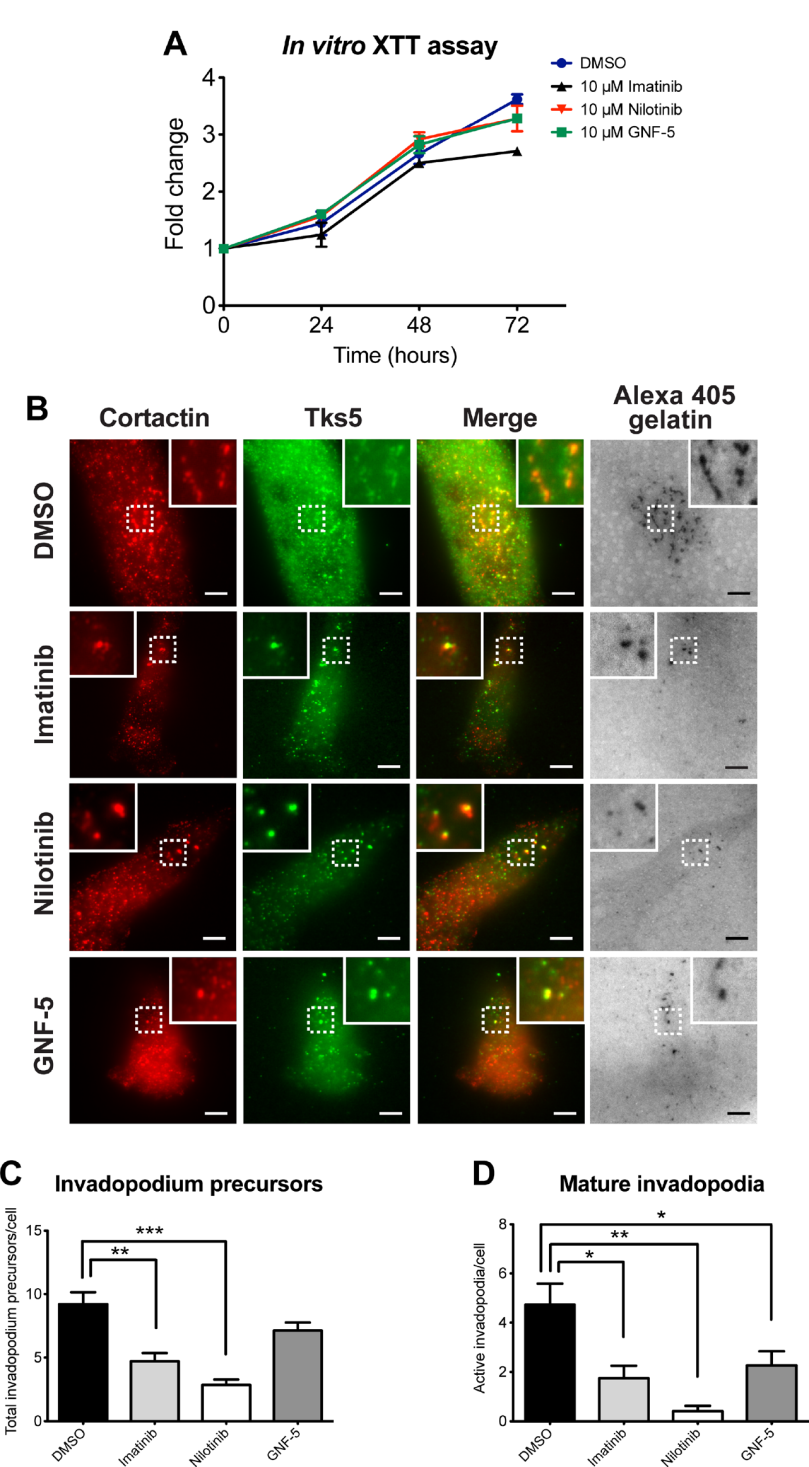
ABL kinase inhibitors affect invadopodium precursor formation and maturation in breast cancer cells (**A**) Viability and proliferation of MDA-MB-231 cells in presence of imatinib (black), nilotinib (red), GNF-5 (green), or DMSO as control (blue) was measured every 24 hours for 72 hours total using the XTT reagent. *n* = 3 independent experiments, each experiment was performed in triplicates. (**B**) MDA-MB-231 cells were pre-treated overnight with 10 μM imatinib, nilotinib, GNF-5, or DMSO as control and then plated on Alexa 405 gelatin, fixed, and labeled for cortactin (red) and Tks5 (green). Boxed regions and insets depict co-localization of cortactin and Tks5 as markers of invadopodia precursors, with Alexa 405 gelatin as a marker of mature invadopodia. Bar, 5 μm. (**C**–**D**) Quantification of invadopodium precursors (C), defined by co-localization of cortactin and Tks5, and mature (active) invadopodia (D), defined by co-localization of cortactin and Tks5 with degradation regions. *n* = 61 (DMSO), *n* = 35 (imatinib), *n* = 30 (nilotinib), *n* = 45 (GNF-5) cells from three independent experiments. ^*^P ≤ 0.05, ^**^P ≤ 0.01, ^***^P ≤ 0.001 as determined by Student’s *t* test. Error bars indicate SEM.

To verify that the concentration of inhibitors used in our experiments (10 μM) does not affect viability or proliferation of MDA-MB-231 cells we used the XTT assay. As demonstrated in Figure 2A, no significant change in proliferation or viability of the kinase inhibitor-treated cells was observed within the period of 72 hours, which is beyond the time used in all *in vitro* experiments described herein.

To examine whether ABL kinase inhibitors regulate the initial assembly of invadopodium precursors, MDA-MB-231 cells were treated with imatinib, nilotinib, GNF-5, or DMSO as control, plated on fluorescently labeled gelatin matrix and labeled for Tks5 and cortactin as invadopodium precursor markers (Figure 2B). Cells that were treated with imatinib or nilotinib, but not with GNF-5, showed a significant decrease in Tks5- and cortactin-positive invadopodium precursors (Figure 2C). Furthermore, a significant decrease in active, matrix-degrading invadopodia was also observed in cells that were treated with imatinib, nilotinib, or GNF-5 (Figure 2D). These data suggest that imatinib and nilotinib affect invadopodium precursor formation by inhibiting tyrosine kinases other than Arg and Abl, while all three inhibitors affect invadopodium maturation and function.

### ABL kinase inhibitors affect cortactin tyrosine phosphorylation at invadopodium precursors

**Figure 3:**
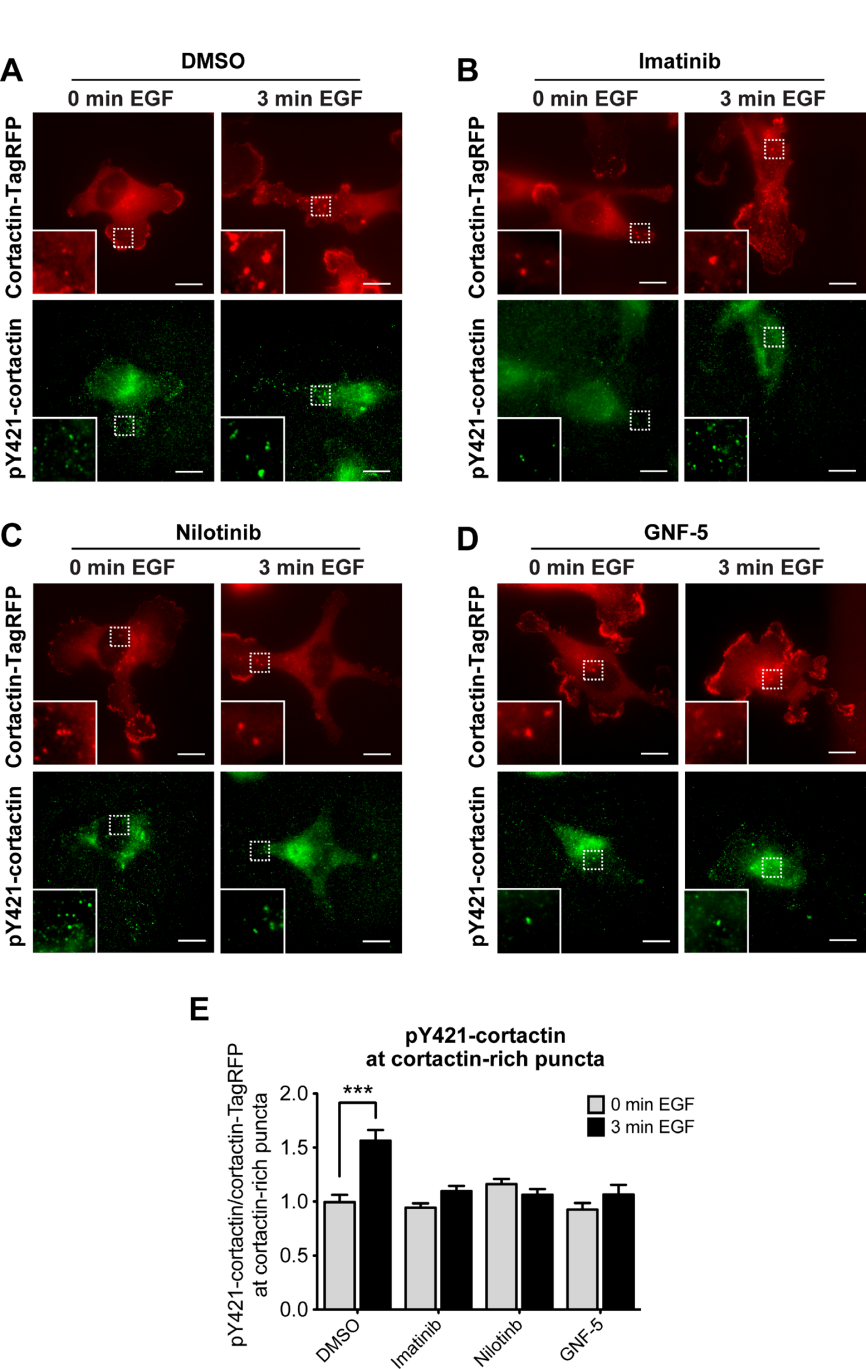
Cortactin tyrosine phosphorylation at invadopodium precursors is significantly reduced by ABL kinase inhibitors MDA-MB-231 cells stably expressing cortactin-TagRFP (red) were pre-treated overnight with 10 μM imatinib (**B**), nilotinib (**C**), GNF-5 (**D**), or DMSO as control (**A**), plated on FN/gelatin matrix, starved, and stimulated with EGF. Cells were fixed and labeled for tyrosine phosphorylated cortactin (anti-pY421-cortactin; green) before (0 min) or after (3 min) EGF stimulation. Bar, 10 μm. (**E**) Quantification of pY421-cortactin/cortactin-TagRFP signal at cortactin-rich puncta. *n* = 203 (DMSO, 0 min), *n* = 176 (DMSO, 3 min), *n* = 134 (imatinib, 0 min), *n* = 207 (imatinib, 3 min), *n* = 186 (nilotinib, 0 min), *n* = 159 (nilotinib, 3 min), *n* = 160 (GNF-5, 0 min), *n* = 184 (GNF-5, 3 min) cortactin-rich puncta from three independent experiments. ^***^P ≤ 0.001 as determined by Student’s *t* test. Error bars indicate SEM.

Maturation and functional activation of invadopodia are dependent on tyrosine kinase-mediated phosphorylation of the invadopodial core protein cortactin [44]. To investigate whether ABL kinase inhibitors impede invadopodial function by controlling cortactin phosphorylation, control and inhibitor-treated cells were stimulated with epidermal growth factor (EGF) to induce invadopodia formation, and labeled with a phosphorylation-specific antibody for cortactin tyrosine Y421 (Figure 3A–3D). Cortactin phosphorylation was increased in cortactin-rich puncta of control cells stimulated with EGF, while no cortactin phosphorylation was detected in imatinib, nilotinib, or GNF-5 treated cells (Figure 3E). Altogether, the results of this experiment suggest that ABL kinase inhibitors regulate the maturation and functional activation of invadopodia by inhibiting cortactin tyrosine phosphorylation in these structures.

### Imatinib, nilotinib, and GNF-5 significantly reduce actin barbed end generation at invadopodia-enriched cellular regions

**Figure 4:**
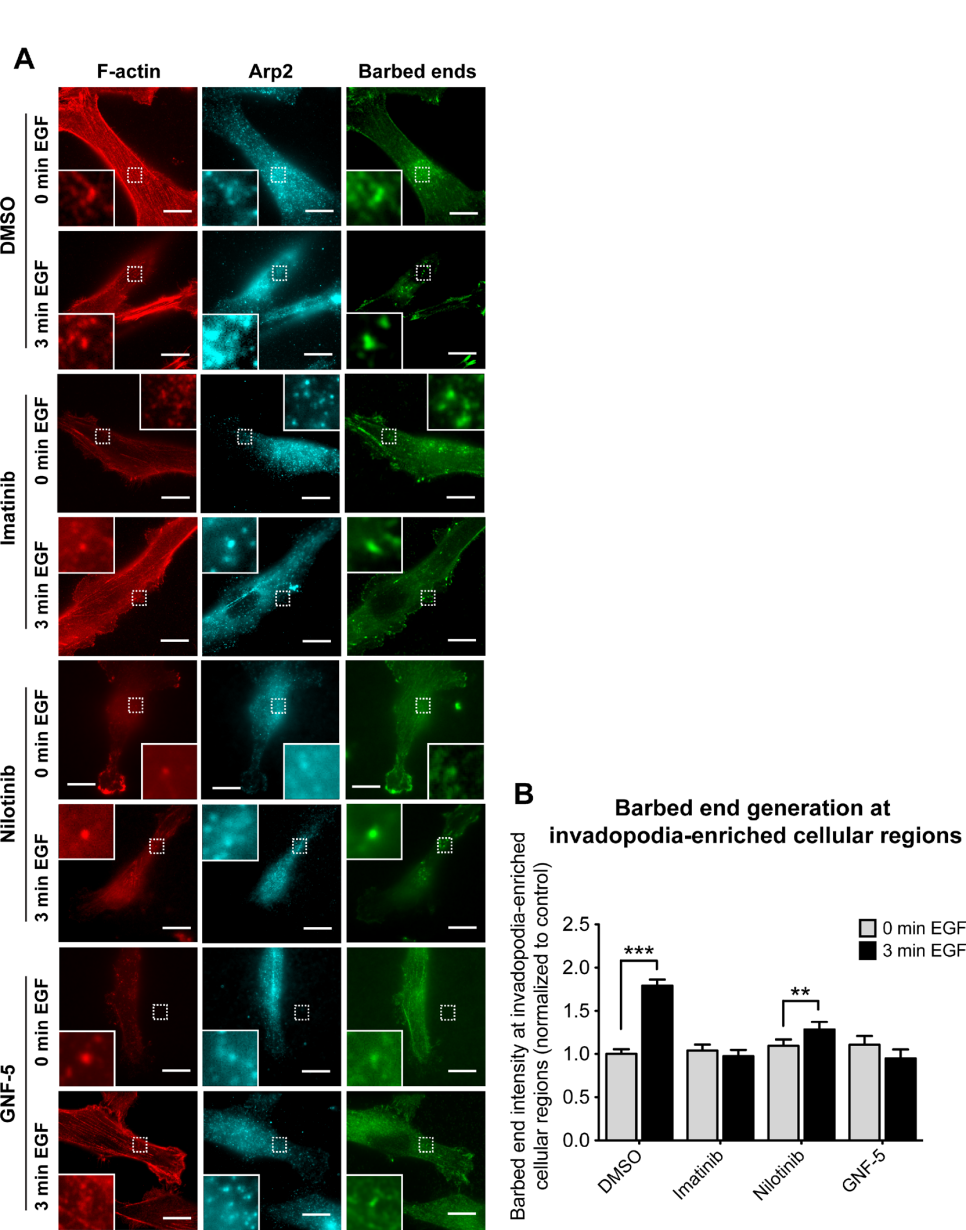
Inhibition of ABL kinases affects barbed end formation in invadopodia of breast cancer cells (**A**) MDA-MB-231 cells were pre-treated overnight with 10 μM imatinib, nilotinib, GNF-5, or DMSO as control, and either left untreated (0 min EGF) or stimulated with EGF for 3 min (3 min EGF). Cells were fixed and labeled for F-actin (red) and Arp2 (cyan) as invadopodia markers, and for biotin-actin (green) as a marker for newly formed barbed ends. Bar, 10 μm. (**B**) Quantification of free actin barbed ends as measured by average biotin-actin intensity at stimulated invadopodia containing F-actin and Arp2. *n* = 77 (DMSO, 0 min), *n* = 54 (DMSO, 3 min), *n* = 50 (imatinib, 0 min), *n* = 54 (imatinib, 3 min), *n* = 28 (nilotinib, 0 min), *n* = 25 (nilotinib, 3 min), *n* = 20 (GNF-5, 0 min), *n* = 24 (GNF-5, 3 min) invadopodia from three independent experiments. ^**^P ≤ 0.01, ^***^P ≤ 0.001 as determined by Student’s *t* test. Error bars indicate SEM.

Tyrosine phosphorylation of cortactin in invadopodia leads to free actin barbed end generation and consequent actin polymerization, which provides the physical force that pushes the invadopod membrane outward into the extracellular matrix and enables the cell to penetrate through it [3, 37]. Because ABL kinase inhibitors significantly reduced cortactin tyrosine phosphorylation in invadopodia in response to EGF, we hypothesized that they might also regulate barbed end generation at invadopodia of inhibitor-treated cells. To test this hypothesis, we stimulated control and inhibitor-treated cells with EGF, to synchronize the generation of free actin barbed ends within regions of cells enriched in invadopodia (Figure 4A). We then quantified free actin barbed end generation specifically within these structures following EGF stimulation. Whereas control cells generated approximately 1.8-fold increase in barbed end intensity in response to EGF stimulation, imatinib and GNF-5 completely disrupted generation of barbed ends at invadopodia-enriched cellular regions, while treatment with nilotinib resulted a mild but significant increase of approximately 1.3-fold in barbed end generation (Figure 4B). The milder effect of nilotinib on barbed end generation at invadopodia may result from its wider specificity towards kinases that are not affected by imatinib or GNF-5 (see Figure 1A). Collectively, these data suggest that imatinib and GNF-5, and to a lesser extent nilotinib, can inhibit EGF-mediated actin barbed end generation at invadopodia of breast cancer cells.

### Inhibition of ABL kinases affects extracellular matrix degradation and 3D directed motility of breast cancer cells

**Figure 5:**
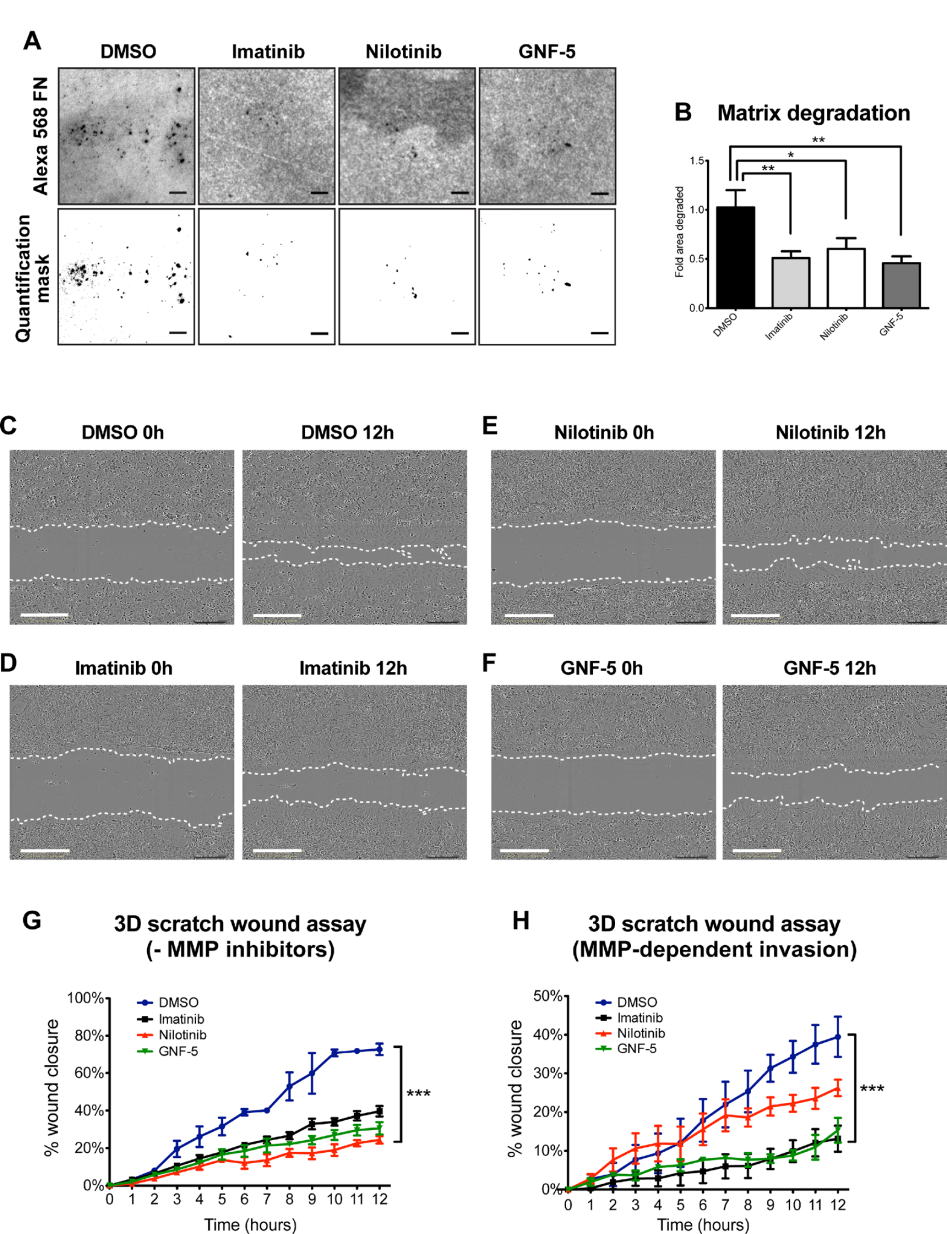
ABL kinase inhibitors decrease extracellular matrix degradation and MMP-dependent 3D directed motility of breast cancer cells (**A**) MDA-MB-231 cells were pre-treated overnight with 10 μM imatinib, nilotinib, GNF-5, or DMSO as control, plated on Alexa 488 FN/gelatin matrix, and allowed to degrade for 24 hours. Shown are representative images (upper panels) and quantification masks (lower panels) of degradation areas. Bar, 10 μm. (**B**) Quantification of matrix degradation by inhibitor-treated cells. *n* = 75 (DMSO), *n* = 71 (imatinib), *n* = 69 (nilotinib), *n* = 64 (GNF-5) fields per group from three independent experiments. ^*^P ≤ 0.05, ^**^P ≤ 0.01 as determined by Student’s *t* test. Error bars indicate SEM. (**C–F**) Representative images from 3D scratch wound assay movies of MDA-MB-231 cells treated with imatinib (D), nilotinib (E), GNF-5 (F), or DMSO (C) as control, at time 0 (0h, left panels) and at 12 hours (12h, right panels). Bar, 300 μm. (**G**) Quantification of 3D directed motility towards a wound in Matrigel containing embedded MDA-MB-231 cells treated with ABL kinase inhibitors (imatinib; black, nilotinib; red, GNF-5; green) or DMSO as control (blue). F = 23.89, ^***^P ≤ 0.001 as determined by two-way ANOVA followed by Tukey post-hoc test. (**H**) Quantification of 3D directed motility towards a wound in Matrigel containing embedded MDA-MB-231 cells treated with ABL kinase inhibitors and normalized to MMP inhibitor-treated values. F = 21.87, ^***^P ≤ 0.001 as determined by two-way ANOVA followed by Tukey post-hoc test. Shown are results from three independent experiments.

At the final stage of their maturation, invadopodia gain the ability to locally degrade the extracellular matrix via recruitment and activation of MMPs [37, 45, 46]. This process allows invasive tumor cells to escape through the basement membrane surrounding the primary tumor, to invade through the stromal extracellular matrix, and to penetrate into blood vessels [47]. To examine the ability of ABL kinase inhibitors to obstruct MMP-mediated extracellular matrix (ECM) degradation by mature invadopodia, control and inhibitor-treated breast cancer cells were plated on fluorescent fibronectin (FN)/gelatin matrix and allowed to degrade it for 24 hours (Figure 5A). Quantification of the degradation area revealed a significant decrease in the ability of inhibitor-treated cells to degrade the matrix comparing to control, DMSO-treated cells (Figure 5B).

Invadopodia acquire their protrusive ability by combining the physical force generated by actin polymerization with the chemical activity of MMP-mediated matrix degradation [48]. Because ABL kinase inhibitors significantly decrease cortactin phosphorylation-mediated actin polymerization as well as matrix degradation in treated invasive cancer cells, we hypothesized that they might also control the ability of the cells to invade through an extracellular matrix barrier. To test this hypothesis, we examined the ability of control and inhibitor-treated cells that were embedded in a 3D Matrigel matrix to invade towards a scratch wound over a period of 12 hours (Figure 5C–5F and Supplementary Movies 1–4). As demonstrated in Figure 5G, cells treated with imatinib, nilotinib, or GNF-5 showed significantly reduced ability to close the gap by invading towards the wound as compared with control, DMSO-treated cells. Interestingly, while imatinib and GNF-5 mainly inhibit proteolysis- and MMP-dependent invasion, nilotinib inhibits both MMP-dependent as well as MMP-independent motility in 3D (Figure 5G–5H).

At the initial stages of cancer metastasis, tumor cell invasion is guided by growth factors and chemoattractants that are secreted by stromal cells in the tumor microenvironment. To test the ability of imatinib, nilotinib, and GNF-5 to inhibit chemotactic invasion of MDA-MB-231 breast cancer cells, we plated serum-starved, inhibitor-treated cells on Matrigel-coated Transwell membranes and measured their ability to invade towards complete medium. As demonstrated in Supplementary Figure 2A–2B, cells that were treated with either one of the inhibitors showed reduced chemotactic invasion towards complete medium, compared to control, DMSO-treated cells. Interestingly, and in agreement with our previous knockdown data [23], no difference was observed between control and inhibitor-treated cells in migration towards complete medium when plated on un-coated Transwell filters (Supplementary Figure 2A, 2C). This result could be explained by the fact that this form of chemotactic migration does not require active, MMP- and invadopodia-dependent motility and was therefore not inhibited by ABL kinase inhibitors. Together, these data suggest that ABL kinase inhibitors can decrease MMP-dependent matrix degradation and consequent chemotactic and 3D matrix invasion, but not matrix-degradation independent chemotactic motility.

### ABL kinase inhibitors suppress 2D random migration as well as MMP-dependent 2.5D invasion of breast cancer cells

**Figure 6:**
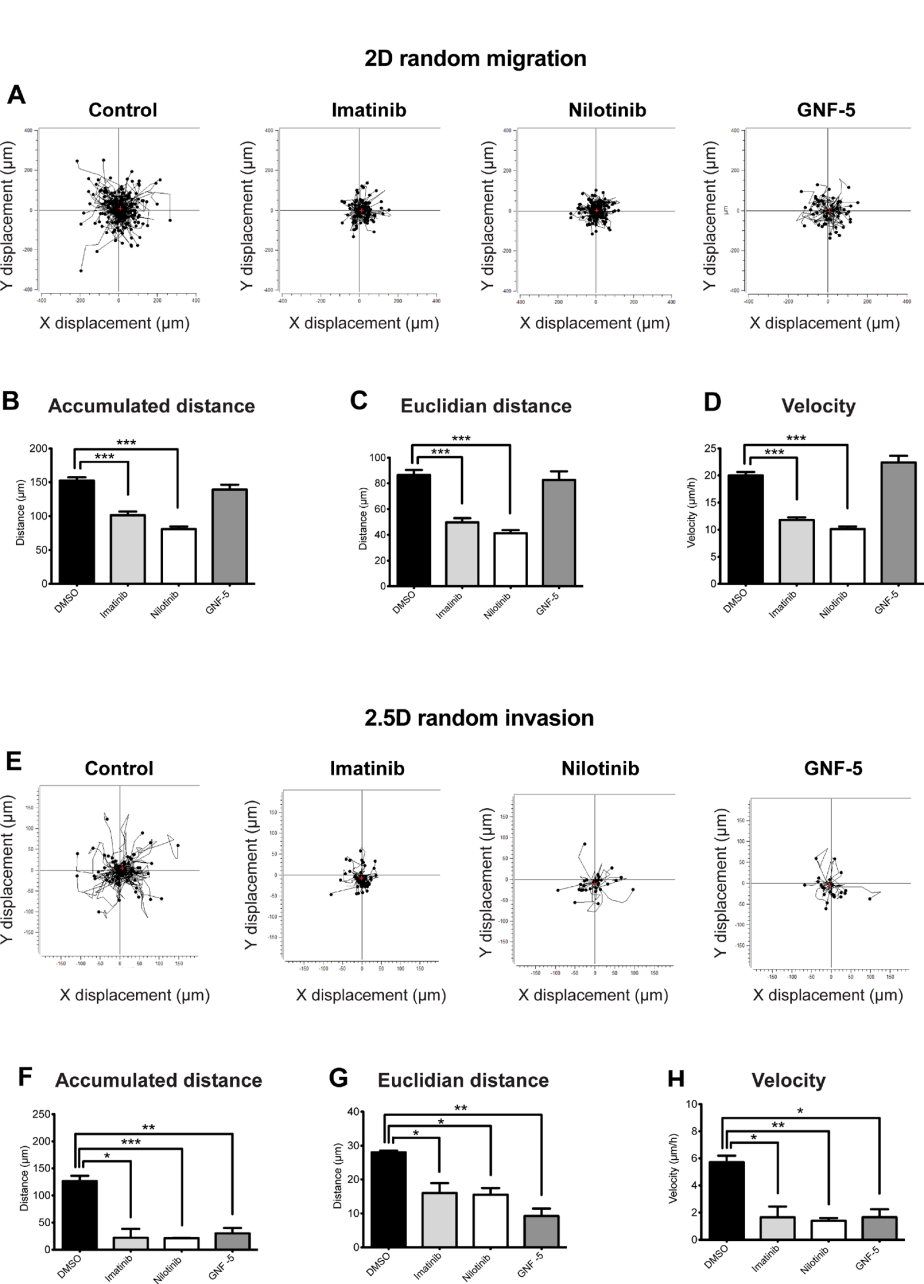
ABL kinase inhibitors suppress 2D random migration and MMP-dependent 2.5D invasion of breast cancer cells MDA-MB-231 cells were plated on fibronectin-coated plates in presence of imatinib, nilotinib, GNF-5, or DMSO as control, placed in 37° C heated chamber and imaged every one hour for a total of 12 hours. (**A**) Trajectory plots demonstrating random cell motility in 2D. (**B–D**) Quantification of motility parameters: accumulated distance (total cell path length) (B), euclidian distance (the shortest distance between the starting point and end point of migration) (C), and velocity (D). *n* = 238 (DMSO), *n* = 191 (imatinib), *n* = 178 (nilotinib), *n* = 56 (GNF-5) cells from three independent experiments. (**E–H**) MDA-MB-231 cells were embedded in Matrigel in presence of imatinib, nilotinib, GNF-5, or DMSO as control, placed in 37° C heated chamber and imaged every one hour for a total of 12 hours. (E) Trajectory plots demonstrating random cell invasion in 2.5D. (F–H) Quantification of accumulated distance (F), euclidian distance (G), and velocity (H) of cell invasion in 2.5D. To control for MMP-dependent invasion, motility of cells in presence of the broad MMP inhibitor GM6001 was measured and subtracted from the total motility values. *n* = 68 (DMSO), *n* = 95 (imatinib), *n* = 85 (nilotinib), *n* = 61 (GNF-5), *n* = 80 (DMSO + MMP inhibitor), *n* = 95 (imatinib + MMP inhibitor), *n* = 58 (nilotinib + MMP inhibitor), *n* = 57 (GNF-5 + MMP inhibitor) cells from three independent experiments. ^*^P ≤ 0.05, ^**^P ≤ 0.01, ^***^P ≤ 0.001 as determined by Student’s *t* test. Error bars indicate SEM.

To gain further insight into the mechanism by which imatinib, nilotinib, and GNF-5 inhibit cell motility and invasion, we examined the behavior of inhibitor-treated cells in 2D versus 2.5D. To follow the behavior of the cells in 2D, control and inhibitor-treated cells were plated on a thin layer of fibronectin and allowed to randomly migrate over it, and the accumulated distance (total cell path length), euclidian distance (the shortest distance between the starting point and end point of migration), and velocity were measured over a period of 12 hours. As demonstrated in Figure 6A–6D, cells treated with imatinib or nilotinib, but not with GNF-5, showed reduced 2D migration over an ECM substrate compared to control, DMSO-treated cells.

To further elucidate the mechanism by which ABL kinase inhibitors affect proteolysis-dependent invasion, inhibitor-treated cells were embedded in Matrigel and allowed to randomly invade through it. To control for MMP-dependent motility, an identical set of cells from each group was plated in presence of the broad range MMP inhibitor GM6001, and values of non-proteolysis dependent motility were subtracted from the total measured values. Interestingly, cells treated with imatinib or nilotinib, as well as cells treated with GNF-5, showed significantly reduced proteolysis-dependent random invasion characteristics in Matrigel (Figure 6E–6H). The different behavior of GNF-5 treated cells in 2D versus 2.5D suggests that, while imatinib and nilotinib inhibit both 2D motility as well as invadopodia- and proteolysis-dependent 3D motility, GNF-5 inhibits MMP-dependent invasiveness only. The difference in inhibition mechanism may rely on the narrow kinase specificity of GNF-5 compared to imatinib and nilotinib.

### Primary tumor growth is not affected by ABL kinase inhibitors

**Figure 7:**
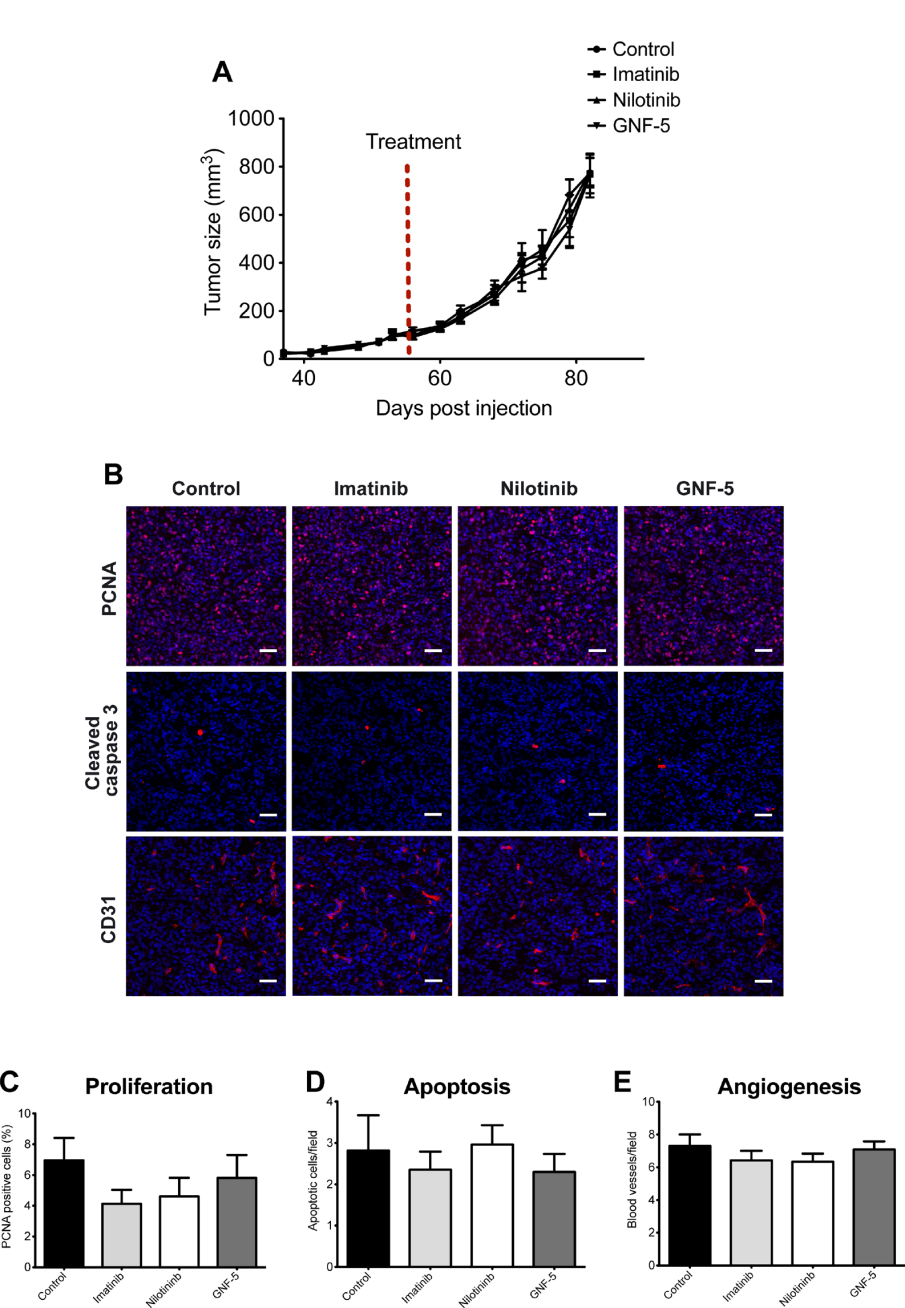
Primary tumor growth is not affected by ABL kinase inhibitors MDA-MB 231/Dendra2 cells were injected into the mammary fat pad of 10-week-old SCID female mice and allowed to grow until the tumor reached the size of 100 mm^3^. At day 56 following injection, mice were treated by oral gavage with vehicle (5% DMSO, 2% hydroxypropyl cellulose, 0.5% Tween-80), 100 mg/kg imatinib, 70 mg/kg nilotinib or 100 mg/kg GNF-5 once a day, 5 days a week, for four weeks. (**A**) Time-dependent tumor growth. Tumor growth was assessed twice a week by measuring two perpendicular diameters and calculating tumor size in mm^3^. Treatment initiation is shown as red dotted line. *n* = 12 (vehicle), *n* = 10 (imatinib), *n* = 12 (nilotinib), *n* = 10 (GNF-5) mice per group from two independent experiments. (**B**) Primary tumors were dissected at the end of experiment and subjected to immunohistochemistry. Representative images of primary tumor sections stained with anti-PCNA (proliferation), anti-cleaved caspase 3 (apoptosis), and anti-CD31 (angiogenesis). (**C**) Quantification of PCNA positive cells (red/pink) normalized to DAPI positive cells (blue). The average percentage of proliferating cells in total cells per field is shown. (**D**) Quantification of apoptotic cells (cleaved caspase 3 positive cells). Shown is the number of apoptotic cells per field. (**E**) Quantification of CD-31 positive blood vessels. For all quantifications, *n* = 50 random fields from 5 tumors per condition. Bar, 100 µm.

Previous publications suggested that ABL kinase inhibitors affect proliferation of cancer cells and consequent tumor metastatic growth [13, 49]. To determine whether inhibitors of ABL kinases affect primary tumor growth *in vivo*, we injected MDA-MB-231 breast cancer cells into the mammary fat pad of immunodeficient mice. Tumor-bearing mice were then treated with imatinib, nilotinib, GNF-5, or vehicle control by oral gavage for four weeks. As demonstrated in Figure 7A, no significant difference in tumor growth or tumor size was observed in mice treated with inhibitors or vehicle control over a period of one month. To gain further insight into the reason for similar tumor size and growth we performed immunofluorescent histological labeling of primary tumor sections from inhibitor- and vehicle control-treated mice. No significant differences were observed in proliferation, apoptosis, or angiogenesis in tumors from the different groups (Figure 7B–7E). In conclusion, treatment with imatinib, nilotinib, or GNF-5 does not affect proliferation, apoptosis, or angiogenesis and consequent primary tumor growth of MDA-MB-231 generated xenograft tumors.

Interestingly, our previous publication suggests that knockdown of Arg in MDA-MB-231 breast tumor cells significantly increases primary tumor size while decreasing invasiveness and metastatic dissemination [8]. This discrepancy between previous data and our current findings using ABL kinase inhibitors could be explained by opposing effects of Arg and Abl in the regulation of breast cancer cell proliferation [8, 50].

### *In vivo* MMP activity, tumor cell invasion are significantly decreased in ABL kinase inhibitor-treated xenograft tumors

**Figure 8:**
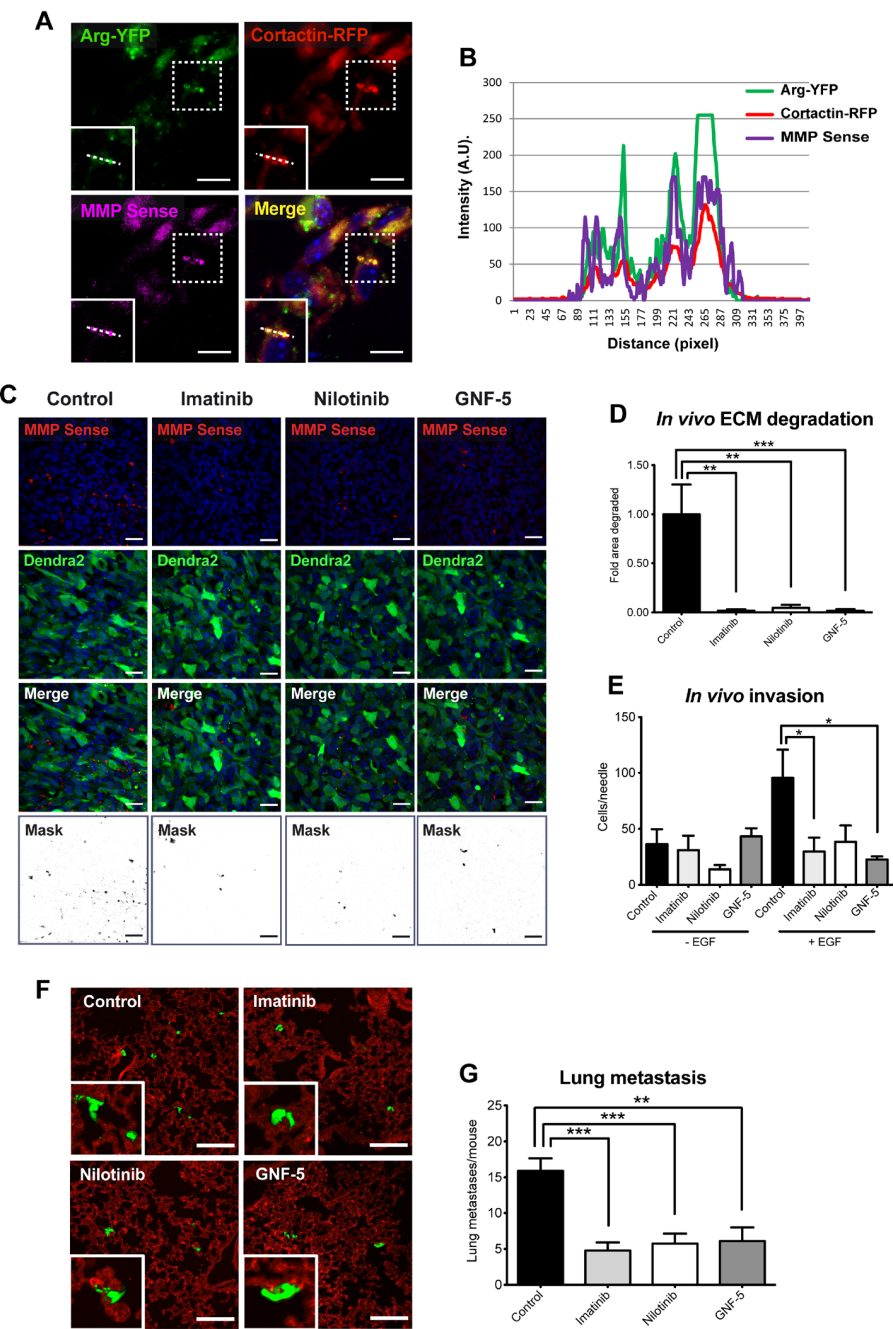
*In vivo* MMP activation, tumor cell invasion, and consequent lung metastasis are significantly decreased in ABL kinase inhibitors-treated xenograft tumors (**A**) Localization of Arg was visualized by confocal imaging using MMP Sense (magenta) in orthotopic xenograft tumors generated from MDA-MB-231 cells stably expressing Arg-YFP (green) and cortactin-TagRFP (red). Shown is co-localization of Arg with cortactin in cellular protrusions that overlap with MMPSense-positive areas. Scale bar, 10 μm. (**B**) Intensity profiles of Arg (green line), cortactin (red line), and MMP Sense (magenta line), illustrating enrichment of these proteins in matrix-degrading protrusions. (**C**) Representative images (top panels) and quantification masks (bottom panel) of degraded area in orthotopic xenograft tumors generated by MDA-MB-231/Dendra2 cells (green) and treated with imatinib, nilotinib, GNF-5, or vehicle as control. ECM degradation was visualized using fluorescently activated MMP Sense (red). Bar, 30 µm. (**D**) Quantification of MMP activity in tumors. *n* = 30 fields (control, imatinib, nilotinib), *n* = 40 fields (GNF-5) from three or four different mice per group, respectively. (**E**) *In vivo* invasion towards EGF was measured in MDA-MB-231/Dendra2 xenograft tumors that were treated with imatinib, nilotinib, GNF-5, or vehicle as above. Total cells were counted following 4’6-diamidino-2-phenylindole (DAPI) staining. *n* = 6–9 needles per group. (**F**) Representative images of lung sections from mice bearing orthotopic tumors generated by MDA-MB-231/Dendra2 cells and treated with imatinib, nilotinib, GNF-5, or vehicle as control as above. Green, tumor cells (Dendra2), red, blood vessels (anti-CD31; endothelial marker). Scale bar, 100 μm. (**G**) Quantification of lung metastases. *n* = 10–12 mice from two independent experiments. ^*^P ≤ 0.05, ^**^P ≤ 0.01, ^***^P ≤ 0.001 as determined by Student’s *t* test. Error bars indicate SEM.

Arg has been shown to localize to Tks5-containing, matrix degrading invadopodia *in vitro* [23] and to control breast cancer metastasis *in vivo* [8], but its association with invadopodia-like tumor cellular protrusions has never been examined. To investigate the cellular localization of Arg within the primary tumor, we examined tissue sections of orthotopic xenograft tumors that were generated from MDA-MB-231 cells expressing Arg-YFP, cortactin-TagRFP, and MMP Sense, a protease-activatable fluorescent *in vivo* imaging agent that is optically silent upon injection and produces a fluorescent signal following cleavage by MMPs. Co-localization of Arg, cortactin, and fluorescently activated MMP Sense was observed in primary tumor sections, supporting enrichment of Arg in matrix-degrading tumor cell protrusions (Figure 8A, 8B).

Our *in vitro* data presented above suggest that ABL kinase inhibitors affect focal MMP-mediated matrix degradation by invadopodia and consequent tumor cell invasiveness. To gain further insight into the *in vivo* inhibition mechanism, and to examine whether ABL kinase inhibitors impede invadopodial MMP-mediated matrix degradation within the primary tumor, we performed the *in vivo* MMP activity assay. Mice bearing fluorescently labeled tumors were treated with imatinib, nilotinib, GNF-5 or vehicle control and injected with MMP Sense before sacrifice. Tumors were then sliced and imaged by confocal microscopy, and the fluorescent signal that was generated by MMP-mediated activity within the primary tumor was quantified. In agreement with our *in vitro* matrix degradation results above, the fluorescent signal, which is directly correlated with MMP activation and consequent ECM degradation, was dramatically decreased in tumors isolated from ABL kinase inhibitor-treated mice compared to tumors from vehicle-treated mice (Figure 8C, 8D).

To examine whether ABL kinase inhibitors affect invasion *in vivo*, in the context of the primary tumor, we used the *in vivo* invasion assay [51]. Immonodeficient female mice bearing equal size MDA-MB-231 originated mammary tumors were treated with imatinib, nilotinib, GNF-5 or vehicle control for four weeks and subjected to the *in vivo* invasion assay. Interestingly, and in agreement with our 2.5D and 3D *in vitro* invasion data, fewer cells succeeded penetrating the needles following EGF stimulation in tumors of mice that were treated with ABL kinase inhibitors compared to tumors of vehicle-treated mice (Figure 8E). Altogether, these observations suggest that ABL kinase inhibitors affect *in vivo* breast tumor cell invasiveness by regulating invadopodia-mediated ECM degradation and tumor cell invasion.

### ABL kinase inhibitors compromise spontaneous lung metastasis in a xenograft mouse model

At the final stage of metastatic dissemination, cancer cells extravasate into the target organ and establish new metastatic colonies. Because ABL kinase inhibitors significantly decreased *in vivo* ECM degradation within the primary tumor as well as *in vivo* invasion from inhibitor-treated tumors, we hypothesized that metastatic dissemination would also be affected. To investigate the ability of ABL kinase inhibitors to suppress spontaneous metastasis to lungs, immunodeficient mice bearing MDA-MB-231 mammary tumors were treated with imatinib, nilotinib, GNF-5, or vehicle control as above, followed by careful examination and quantification of lung metastases at the end of treatment. As demonstrated in Figure 8F–8G, mice that were treated with ABL kinase inhibitors exhibited significantly fewer lung metastases than vehicle-treated mice bearing equal size tumors. Collectively, these data suggest that ABL kinase inhibitors can suppress *in vivo* breast tumor metastatic dissemination.

### Proteogenomic database analysis of patient breast tumors reveals increase in Arg and cortactin across all breast tumor subtypes

**Figure 9:**
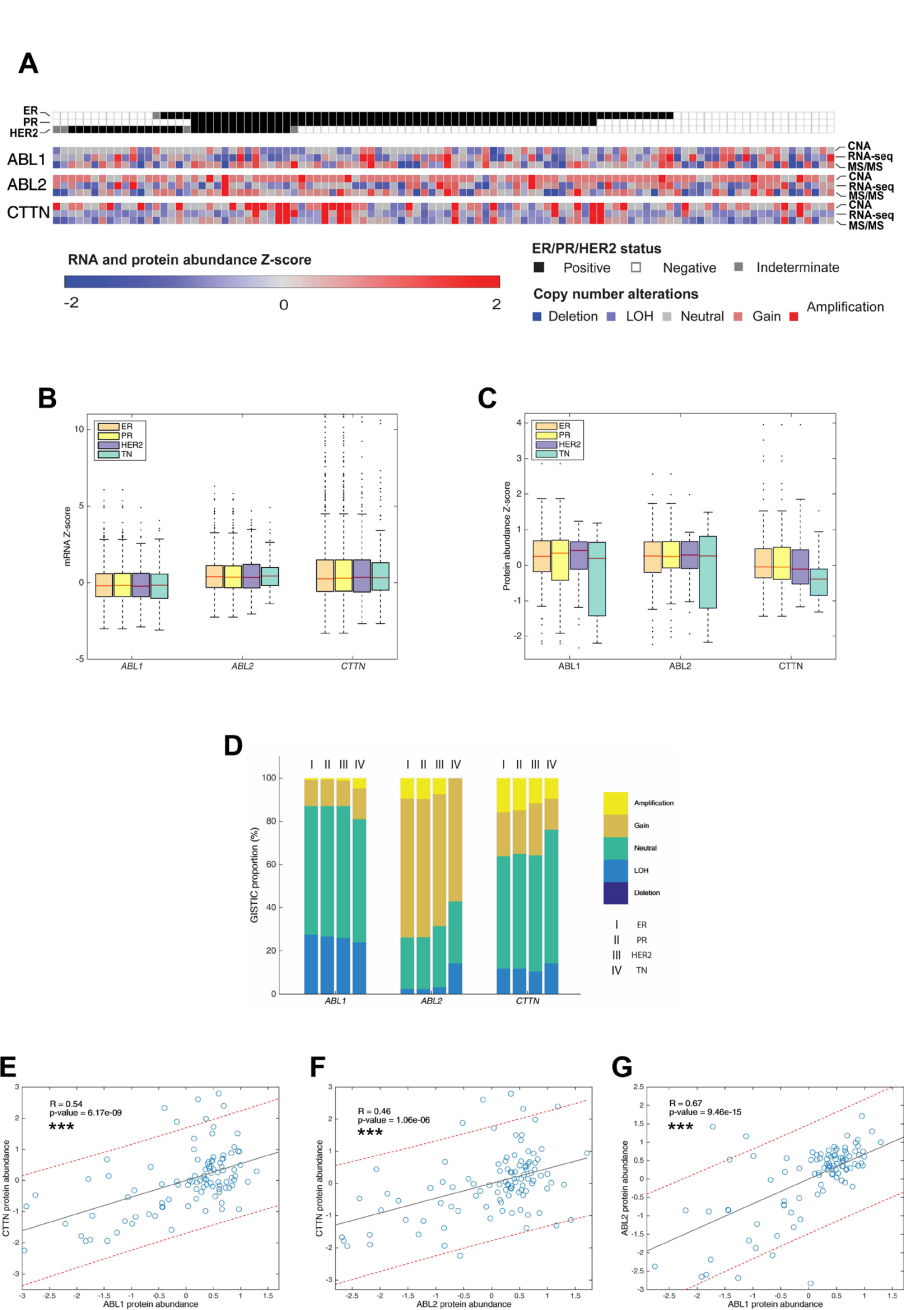
Proteogenomic database analysis of patient breast tumors (**A**) Heat map of copy number alteration (CNA), mRNA expression, and protein expression of Abl (ABL1), Arg (ABL2) and cortactin (CTTN) across 102 tumor samples based on the CPTAC database. ER, estrogen receptor positive; PR, progesterone receptor positive; HER2, HER2 positive, TN, triple negative. GISTIC, mRNA and protein abundance z-scores are shown for each sample. (**B**) mRNA expression analysis of 1,905 samples originated from TCGA with different hormone receptor statuses. (**C**) Comparison of protein abundance in 102 samples from different hormone receptor statuses. The boxes in B and C is delimited by the lower and upper quartile, the horizontal red line indicates the sample median and whiskers extend to the most extreme point which is no more than 1.5 times the interquartile range from the box. Outliers are shown as points beyond boxplot whiskers. *P* values were calculated using two-tailed Student’s *t*-test. (**D**). GISTIC analysis of 1,905 samples originated from TCGA with different hormone receptor statuses. (**E**–**G**) Pearson correlation between ABL1-CTTN, ABL2-CTTN and ABL1-ABL2 protein abundances. ^***^P ≤ 0.001 as determined by Student’s *t* test.

To assess the involvement of ABL kinases and cortactin in metastatic dissemination in breast cancer patients, we integrated DNA, RNA, and protein expression data of breast cancer tumors from TCGA and compared them for hormone- and HER2-receptor status. A comparison of the percentage of somatic mutations in ABL1, ABL2, and CTTN genes in a dataset of 1,905 samples revealed a very low mutation rate for each of the three genes (0.61%, 0.33%, and 0.17% for ABL1, ABL2, and CTTN, respectively), suggesting that mutagenesis does not play a major role in metastasis promotion by these genes. Next, we analyzed expression data of breast tumor samples for copy number alterations (CNA), mRNA expression, and protein expression. A comparison of these three parameters demonstrated similar distribution among all hormone- and HER2-receptor statuses (Figure 9A–9C). GISTIC analysis for amplifications and deletions depicted copy number gain and amplification in ABL2 and CTTN, where the majority of tumor samples had gain or amplification in ABL2 (Figure 9D). These data suggest that Arg and cortactin are amplified in a significant fraction of breast tumors and across all receptor statuses.

**Figure 10:**
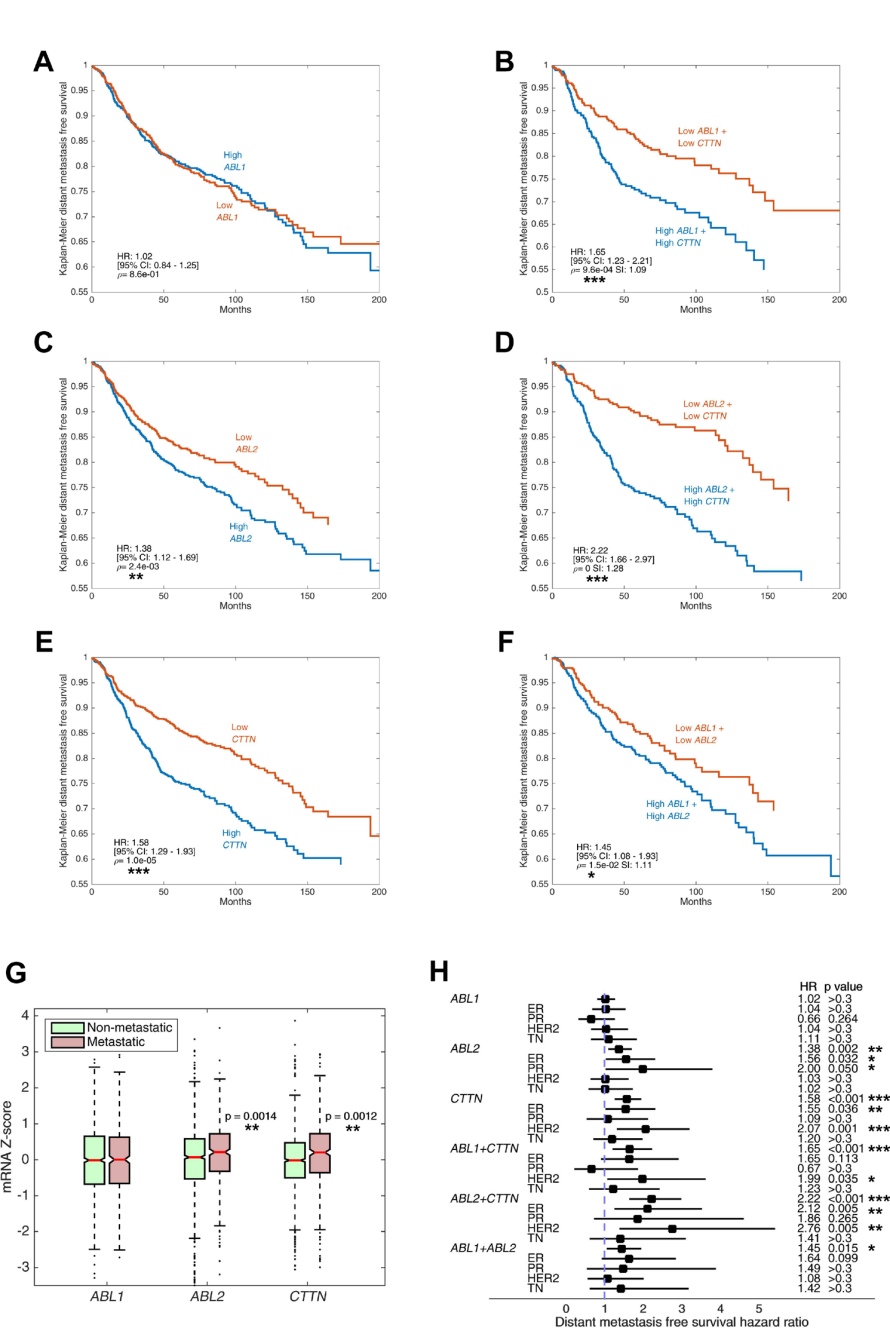
Increased expression of *ABL2* and *CTTN* genes, but not *ABL1*, is associated with breast cancer metastasis and poor patient prognosis (**A–F**) Kaplan–Meier curves of distant metastasis-free survival (DMFS) in 1,650 breast cancer cases. Microarray data (Affymetrix) were obtained from the NCBI Gene Expression Omnibus (GEO) data repository. Tumor samples were split into high and low expressing groups based on mRNA gene expression with Z-score cut-off value 0. Shown are DMFS survival curves for ABL1, ABL2, CTTN and combinations thereof. *P* values were calculated by log-rank test, hazard ratio (HR) and 95% confidence interval (CI) are shown. Synergy index (SI) is indicated where two genes are evaluated. (**G**) mRNA expression levels of ABL1, ABL2 and CTTN in metastatic versus non-metastatic breast cancer patients is shown in boxplots. Metastasis was defined as occurrence of distant metastasis event within the follow-up period. The box is delimited by the lower and upper quartile, the horizontal red line indicates the sample median, the notches represent the 95% CI, and whiskers extend to the most extreme point which is no more than 1.5 times the interquartile range from the box. Outliers are shown as points beyond boxplot whiskers. (**H**) DMFS hazard ratio of ABL1, ABL2 and CTTN and their combinations, stratified by hormone receptor status. Squares indicate hazard ratios and horizontal bars correspond to 95% confidence intervals. ^*^P ≤ 0.05, ^**^P ≤ 0.01, ^***^P ≤ 0.001 as determined by Student’s *t* test.

We next analyzed patient breast tumor databases for a correlation between protein abundance levels of Abl, Arg, and cortactin. Significant Pearson correlation was observed between Abl (ABL1) and Arg (ABL2), with a more moderate correlation between cortactin (CTTN) and Abl or cortactin and Arg (Figure 9E–9G). The correlation between an increase in ABL1 and ABL2 protein levels, which is not accompanied by a similar association in distant metastasis free survival (DMFS) (Figure 10A–10F) suggests contrasting roles for the two kinases in promoting breast cancer metastasis. Further Pearson correlation analysis between mRNA to protein levels for Abl, Arg, and cortactin revealed insignificant correlation in Abl and Arg, whereas a good correlation was observed for cortactin (Supplementary Figure 3A–3C). These observations suggest that while cortactin expression levels are mainly regulated at the transcription level, the expression of Arg and Abl are mostly regulated by a post-transcriptional mechanism [52, 53]. Collectively, these analyses suggest that Arg and cortactin, but not Abl, are amplified in metastatic breast tumors in both mRNA and protein levels and independently of their hormone or HER2 receptor status.

### Increased expression of *ABL2* and *CTTN* genes, but not *ABL1*, is associated with breast cancer metastasis and poor patient prognosis

To assess the involvement of ABL kinases and cortactin in metastatic dissemination and the clinical potential of their inhibition, we analyzed microarray expression levels of 1,650 breast cancer samples. Kaplan–Meier plots of DMFS in the total population showed significant correlation between poor DMFS and high mRNA levels of *ABL2* and *CTTN*. Strikingly, the combined effect of *ABL2* and *CTTN* overexpression considerably reduced DMFS values with a synergy index (SI) of 1.28, suggesting that Arg and cortactin act synergistically to promote breast cancer metastasis. Conversely, mRNA expression of *ABL1* was not associated with a significant change in DMFS and did not present marked SI with either *ABL2* or *CTTN* (Figure 10A–10F). As an alternative approach for evaluating the correlation between metastatic dissemination and overexpression of *ABL1*, *ABL2*, or *CTTN*, groups were divided by distant metastasis occurrence regardless of time-to-event. In agreement with our above data, the mRNA levels of *ABL2* and *CTTN*, but not of *ABL1,* were significantly higher in metastatic tumors compared with tumors that did not metastasize (Figure 10G).

Next, we performed patient survival analysis stratified by hormone receptor status. Our analysis revealed significant correlation of poor DMFS with overexpression of *ABL2* in estrogen receptor (ER) positive and progesterone receptor (PR) positive tumors, and with *CTTN* in ER positive and HER2 positive tumors. The combined effect of overexpression of both *ABL2* and *CTTN* presented increased hazard ratio in all hormone or HER2 receptor statuses, albeit insignificant in PR and triple negative (TN) tumors (Figure 10H). The apparent involvement of Arg and cortactin in distant metastasis across all hormone receptor statuses suggests that they have a synergistic general role in promoting breast cancer metastasis. Nevertheless, the hormone status specific behavior of ABL2 alone suggests that the mechanism of promoting metastasis in HER2 and TN tumors may be dependent on activation of the kinase by upstream signaling and not by its overexpression. We next sought to determine a connection between mRNA expression levels of *ABL1*, *ABL2*, and *CTTN* in breast tumors to overall survival (OS) and disease-free survival (DFS) of breast cancer patients. Careful analysis of RNA sequencing and microarray data from 1,905 breast tumor samples revealed that, although some cases were characterized with increased hazard ratio, there was no significant change in either DFS or OS in any of the genes in all hormone receptor status groups (Supplementary Figure 4). Lack of correlation between *ABL1*, *ABL2*, or *CTTN* expression and DFS or OS could be explained by the different components that are included in these specific analyses, such as local recurrence of the primary tumor, pre-existing metastasis or death of patients from causes that are not related to the disease. Together, these observations suggest that overexpression of *ABL2* and *CTTN*, either individually or synergistically, is correlated with breast cancer metastasis and poor patient prognosis.

## DISCUSSION

Accumulating evidence suggest a link between the ability of cancer cells to form invadopodia in culture and their invasive and metastatic potential *in vivo* [8–11, 54]. Here, we demonstrate that ABL kinase inhibitors significantly reduce invadopodium precursor formation and maturation in breast cancer cells. Treatment with ABL kinase inhibitors significantly reduced cortactin tyrosine phosphorylation in invadopodia and consequent actin polymerization, matrix degradation, and 3D tumor cell invasion. These effects were correlated with the ability of ABL kinase inhibitors to significantly decrease the *in vivo* MMP-mediated matrix degradation and invasiveness of cancer cells within the primary tumor, and with decreased pulmonary metastasis of breast cancer cells in a xenograft mouse model. Given that invadopodia formation and function is related to the *in vivo* invasive and metastatic capacity of cancer cells, and based on the correlation between inhibition of invadopodial functions *in vitro* and inhibition of invasiveness and metastasis *in vivo* by ABL kinase inhibitors, it is reasonable to assume that invadopodia could be considered as a marker for predicting patient outcome following anti-metastasis therapy.

Along these lines, a comparison between 2D motility and 2.5D or 3D invasion of breast cancer cells in presence of ABL kinase inhibitors revealed that while imatinib and nilotinib inhibit both modes of motility, GNF-5 could only inhibit 2.5D and 3D invasiveness of breast cancer cells. Based on the specificity range of ABL kinase inhibitors, we suggest that all three inhibitors impede 3D invadopodia-mediated invasion by inhibiting Arg kinase, while imatinib and nilotinib inhibit 2D migration by targeting other tyrosine kinases. The correlation of our *in vitro* 2.5D and 3D invasion results and the *in vivo* metastasis data suggests that 2.5D or 3D invasion, but not 2D motility, may be considered as a method for predicting the invasive behavior and the response of breast cancer cells and tumors to potential anti-metastatic therapies.

ABL kinases are transiently activated by growth factor receptors or by integrin engagement, leading to cytoskeletal reorganization that is required for lamellipodial protrusion, membrane dorsal ruffles, as well as cell migration and invasion. Regulation of this cytoskeletal dynamics is mediated by binding and phosphorylation of target proteins, which are also associated with invadopodia formation and maturation, such as N-WASp, WAVE, Nck1/2, MENA/Vasp and others [12, 55, 56]. Whether and how similar interactions exist between ABL kinases and their substrates or interactors in invadopodia, and can ABL kinase inhibitors block cancer metastasis by affecting these interactions in invadopodia is a subject for future investigation.

Chevalier and colleagues [57] have shown that imatinib and nilotinib decrease both matrix degradation and invasion in MDA-MB 231 and MDA-MB 468 cells by using low drug concentrations or exposure times. However, cell lines that displayed higher levels of ABL kinase activity, such as BT-549 and Src-transformed fibroblasts, presented contrasting results. In addition, nilotinib treatment of MCF-7 [58] resulted in increased VEGF expression *in vitro*, especially in low doses, and treatment *in vivo* resulted in lower vessel maturation but higher vessel density albeit without concurrent significant change in tumor volume. These results suggest that cancer cell lines with higher ABL activity or desmoplastic response should be counter-balanced by higher drug concentrations. This is also reinforced in a study by Litz *et al.* [59], in which higher but clinically relevant imatinib concentrations block VEGF expression in small cell lung cancer cells.

Numerous studies have demonstrated the anti-proliferative or anti-angiogenic potential of ABL kinase inhibitors in several neoplasms, including breast [60, 61], ovarian [62] and lung [59] cancer cells in an ABL, PDGFR or c-Kit specific manner. This suggests that cytotoxic effects may occur in cancer cell overexpressing oncogenes in the target range affected by imatinib or nilotinib but not in cells lacking the genes thereof [62]. Conversely, ABL kinase inhibitors did not produce prominent anti-proliferative activity in our current study. The inconsistency in different breast cancer cell lines could be explained by different assays and the conditions used therein. However, in coherence with our *in vivo* results, no significant change in proliferation, apoptosis or angiogenesis was observed following prolonged administration of ABL kinase inhibitors in the xenograft mouse model. These results are also supported by the failure in blocking tumor progression in two phase II clinical trials using imatinib combined with monotherapies and compared to monotherapies alone in the treatment of patients with metastatic disease [63, 64].

In contrast to our findings in breast cancer, a recent publication demonstrated that glioblastoma multiforme (GBM) cell lines treated with imatinib and nilotinib show increased invasion, however the results were not corroborated with an *in vivo* model [65]. Nevertheless, the study showed that the pro-invasive activity is mediated through p130Cas and FAK signaling where knockdown of Abl or Arg alone or together had no effect on the mediating genes. Despite the proposition that imatinib and nilotinib induce invasiveness in GBM, the results of multi-center phase III clinical trial for patients with recurrent GBM indicated that there are no clinically meaningful differences (improvement or deterioration) between monotherapies or combination therapies with imatinib [59]. Together, the results indicate that ABL kinases may not play a major role in the invasiveness of some neoplasms such as GBM, which is supported by the reduced Arg expression levels in GBM tissues compared to breast cancer, according to the Human Protein Atlas [66]. In addition, CML exposure to imatinib was reported by several groups to induce chemoresistance, which is mediated by stromal cells via secreted factors such as IL-6 and IL-8 [28, 67, 68]. To avoid ABL-independent chemoresistance and in accordance with the emerging role of IL-6 and IL-8 in promoting invasiveness [69], one could use a combination therapy strategy.

Our proteogenomic patient database analysis revealed a uniform distribution of overexpression of Arg kinase and its substrate cortactin in both mRNA and protein levels and across all hormone- and HER2-receptor statuses. Furthermore, we have shown here that overexpression of Arg and cortactin correlates with increased metastasis and poor patient prognosis in breast cancer patients. Together, these findings establish a prognostic value for Arg and cortactin expression levels as predictors for developing distant metastasis and may be used for both risk assessment and therapeutic intervention.

In contrast to the increase in Arg mRNA expression in metastatic breast cancer patients, no increase in Abl mRNA expression either alone or synergistically with cortactin was observed in correlation with metastatic disease. This observation goes along with previous publications suggesting that Arg, but not Abl, has a role in cortactin phosphorylation-mediated invadopodia maturation and consequent *in vivo* invasiveness of breast cancer cells [8, 23]. Despite no correlation between mRNA and protein expression, a significant correlation in protein expression levels of Arg and Abl was observed in breast cancer tumors, suggesting that Arg and Abl may fulfill different roles in the tumorigenic process. Indeed, Abl and Arg may have distinct roles in regulation of breast cancer cell proliferation. Knockdown of Abl in MDA-MB-231 breast cancer cells and human mammary epithelial cells overexpressing the nuclear protein geminin significantly reduced orthotopic mammary tumor growth in a xenograft mouse model [50]. In contrast, knockdown of Arg in the same cells results enlarged tumor size due to increased tumor cell proliferation [8]. These results suggest that Abl and Arg may have opposing roles in tumor cell proliferation. This is supported by our observation that no change in either cell proliferation or primary tumor growth was observed following treatment with ABL kinase inhibitors, which inhibit both kinases. Based on these observations, we suggest that Arg and Abl fulfill different roles in the tumorigenic process. While Arg regulates invadopodia-mediated cancer invasiveness and metastatic dissemination, Abl might be involved in earlier stages of the tumorigenic process, such as cell proliferation and survival. These two processes could play a complementary role in early stages of tumor development, as increased proliferation of tumor cells could lead to increased mutation rate and consequent accumulation of pro-metastatic cellular changes.

To date, several clinical trials have been conducted using imatinib in breast cancer patients (https://clinicaltrials.gov). While all of these experiments exclusively evaluated drug safety or drug efficacy in primary tumor regression, none of these evaluated inhibition of breast cancer metastatic dissemination. We propose here that the ABL kinase inhibitors imatinib, nilotinib, or GNF-5 could be considered for inhibition of breast cancer metastasis. Interestingly, all three inhibitors were similarly efficient in inhibiting breast cancer metastasis in a xenograft mouse model, suggesting that inhibition is mainly derived from decreasing Arg kinase activity and consequent invadopodia-mediated invasiveness. While imatinib and nilotinib are already clinically approved for the use in leukemia, and have some advantage because of their broader inhibition range that may lead to inhibition of alternative pathways, GNF-5 is the most selective and could theoretically lead to a reduced side effects load. In addition, GNF-5 can be applied to imatinib or nilotinib resistant tumors or in combination to avoid resistance; this could be crucial when planning chronic treatment for breast cancer patients for long periods of time.

The ability of breast cancer cells to disseminate by formation and activation of invadopodia is most likely independent of their tumorigenicity, as knockdown or inhibition of several essential invadopodia components has little effect on cell growth *in vitro* or primary tumor growth *in vivo* [10, 11, 70]. Therefore, invadopodia targeted inhibitors may not be effective in blocking the growth of secondary micro- or macro-metastases that have already been established. Nevertheless, such inhibitors could be used to block further metastatic spread and might be more effective when used in combination with cytotoxic chemotherapy.

## MATERIALS AND METHODS

### Antibodies and reagents

For immunofluorescence, anti-cortactin (ab-33333) and anti-PCNA were obtained from Abcam; anti-Arp2 (H-84) (SC-15389) and anti-Tks5 (FISH M-300) (SC-30122) were obtained from Santa Cruz Biotechnology; anti-pY421-cortactin (C0739) was obtained from Sigma-Aldrich; anti-CD31 was obtained from BD Biosciences; anti-cleaved caspase 3 was obtained from Cell Signaling Technology. Rhodamine-labeled phalloidin and Alexa Fluor-conjugated secondary antibodies were obtained from Molecular Probes (Thermo Fisher Scientific). MMP Sense 645 FAST fluorescent imaging agent (NEV10100) was obtained from Perkin-Elmer. Imatinib mesylate (STI-571) (HY-50946), nilotinib (HY-10159), and GNF-5 (HY-15738) were purchased from MedChem Express.

### Constructs and cell lines

MDA-MB-231 human breast adenocarcinoma cells were obtained from American Type Culture Collection (ATCC) and cultured in DMEM/10% FBS. MDA-MB-231 cells stably expressing Dendra2 (MDA-MB-231/Dendra2 cell line) or cortactin-TagRFP were previously described [9, 44]. Stable cell lines containing both Arg-YFP and cortactin-TagRFP were generated by infecting MDA-MB-231 cells with viral sup that was collected from GP2 packaging cells (Clontech) that were transfected with pLXSN-Arg-YFP [23] followed by selection with G418. Arg-YFP cells were then infected with viral sup containing pQCTK-cortactin-TagRFP and selected with Hygromycin.

### Structural modeling

The kinase domain crystal structures of Abl complexed with imatinib and GNF-2 (PDB ID: 3K5V), Abl complexed with nilotinib (PDB ID: 3CS9) and Arg complexed with imatinib (PDB ID: 3GVU) were structurally aligned. GNF-5 was flexibly aligned over GNF-2 using largest common Bemis-Murcko scaffold in Schrödinger’s Maestro 11.0 [71].

### XTT assay

MDA-MB-231 cells were re-suspended to a concentration of 3 × 10^4^ cells/ml in DMEM/10% FBS and plated at 100 μl/well in triplicates in flat bottom 96 well microtiter plates. Following overnight incubation, inhibitors or DMSO as control were added to a final concentration of 10 μM and cells were followed every 24 hours for 72 hours total. Activated XTT solution was prepared according to the manufacturer’s instructions (Biological Industries) and added to cells. Cells were then incubated at 37° C for 4 hours. Specific absorbance was calculated using the formula:

Specific absorbance = A_470_ nm (Test) – A_470_nm (Blank) - A_660_ nm (Test).

### Invadopodia assay and epi-fluorescence microscopy

The invadopodium precursor formation assay was performed as previously described [33]. Briefly, gelatin was conjugated to Alexa 405 dye (Thermo Fisher Scientific). MatTek dishes were treated with 1 N HCl and coated with 50 μg/ml poly-L-lysine. A 0.2% gelatin solution was prepared in PBS, and a 1:40 mixture of Alexa 405-labeled gelatin/unlabeled gelatin was warmed to 37° C before addition to the poly-L-lysine coated plates. Gelatin was cross-linked with 0.01% glutaraldehyde followed by quenching with 5 mg/ml sodium borohydride. MDA-MB-231 cells were pre-treated with imatinib, nilotinib, GNF-5 (10 μM each) or DMSO control for 16 hours. 150,000 pre-treated cells were plated on Alexa 405-labeled gelatin plates for 4 hours and then fixed in 3.7% paraformaldehyde. Cells were permeabilized with 0.1% Triton X-100, blocked with 1% FBS, 1% BSA in PBS, and labeled with anti-Tks5 and anti-cortactin. Images were acquired using an inverted fluorescent microscope (Leica AF6000; 63×, NA 1.4, oil objective, Leica LAS AF acquisition software) equipped with an ORCA-Flash 4.0 V2 digital CMOS camera (Hamamatsu). Invadopodium precursors were identified as Tks5- and cortactin-rich punctate structures found in the ventral plane of the cell, that do not co-localize with degradation areas, whereas mature invadopodia were identified as Tks5 and cortactin-rich puncta that co-localize with degradation areas at the ventral plane of the cell.

### Confocal microscopy and X-Z view of invadopodia

MDA-MB-231 were plated on Alexa 405-labeled gelatin for 4 hours, fixed, and fluorescently labeled with anti-Tks5 and anti-cortactin as above. Z-stacks of 0.7 μm were acquired using a confocal microscope (Zeiss LSM 780; 63×, NA 1.4, oil objective, ZEN black edition acquisition software). The X-Z orthogonal views were generated in FIJI using Reslice function (Supplementary Figure 1).

### Immunofluorescence analysis of cortactin tyrosine phosphorylation at invadopodium precursors

24 hours prior to each experiment, cells were plated on fibronectin/gelatin unlabeled matrix and allowed to attach for 8 hours. Cells were then starved in DMEM/0.5% FBS for 16 hours in presence of inhibitors or DMSO control, and stimulated with 2.5 nM EGF for 3 minutes or left un-stimulated (0 min EGF). Cells were fixed and stained with mouse phospho-specific cortactin antibody (pY421). To image invadopodia Z-plane we focus on the ventral surface of the cell where leading edge containing cortactin is clearly focused. We then analyze the pixels corresponding to invadopodia dots, not the whole cell. The intensity (mean grey value (mgv) minus background) of cortactin-TagRFP and pY421-cortactin at invadopodium precursors was quantified, and the pY421-cortactin/cortactin-TagRFP ratio was calculated as a measure of tyrosine phosphorylated cortactin at cortactin-rich puncta. Data were normalized to resting cells (0 min EGF) and presented as relative fold change in cortactin tyrosine phosphorylation.

### Barbed end formation assay

The barbed end assay was performed using biotin-conjugated actin as previously described [72]. In brief, cells were starved, stimulated with 2.5 nM EGF and permeabilized with a permeabilization buffer (20 mM Hepes, pH 7.5, 138 mM KCl, 4 mM MgCl_2_, 3 mM EGTA, 0.2 mg/ml saponin, 1 mM ATP, and 1% BSA) containing 0.4 μM biotin-conjugated muscle actin (AB07, Cytoskeleton Inc.) for 1 minute at 37° C. Cells were fixed in 3.7% paraformaldehyde for 5 minutes, blocked in PBS containing 1% FBS, 1% BSA and 3 μM un-labeled phalloidin (Molecular Probes, P3457), then labeled with FITC anti-biotin (200-092-211, Jackson ImmunoResearch Laboratories) to visualize barbed ends, and with rhodamine-phalloidin (Molecular Probes, R415) and Arp2 to identify regions of cells rich in invadopodia. To image newly formed barbed ends, which form at invadopodia at the ventral side of the cell, we first focused on Arp2 and actin which are localized at the cell leading edge. We then analyze only the pixels corresponding to invadopodia dots.

The barbed end intensity at invadopodia-rich regions was quantified by measuring mgv at invadopodia-rich regions minus mgv of the background. Data was normalized to the control condition for each experiment.

### *In vitro* matrix degradation assay

The *in vitro* matrix degradation assay was performed as previously described [23]. Briefly, MatTek dishes were treated with 2.5% gelatin/2.5% sucrose, cross-linked with 0.5% glutaraldehyde, treated with 10 μg/ml of fluorescently-labeled fibronectin (Alexa 568; Invitrogen, Thermo Fischer Scientific) and then with 1 mg/ml NaBH4 in PBS. 125,000 MDA-MB-231/Dendra2 cells were plated on the fibronectin/gelatin matrix in presence of inhibitors or DMSO control and allowed to degrade for 24 hours. Cells were then fixed in 3.7% paraformaldehyde and random fields were imaged using an inverted fluorescent microscope (Leica AF6000; 40×, NA 1.3, oil objective). ECM degradation was analyzed by quantifying the average degraded area in pixels per field using ImageJ.

### 3D scratch wound assay

96 well Image Lock microtiter plates (Essen Bioscience) were coated with 100 μg/ml Matrigel (Corning; cat # 354234). Following overnight incubation at 37° C, MDA-MB-231 cells were plated in a final concentration of 350,000 cells/ml and allowed to adhere for 12–16 hours. Following wounding of the bottom Matrigel-cells layer a top layer of Matrigel was added to a final concentration of 2 mg/ml and allowed to polymerize for 30 minutes at 37° C. Inhibitors or DMSO were then added in DMEM/10%FBS to a final concentration of 10 μM, with or without the MMP inhibitor GM6001 (25 μM). Plates were placed in a 37° C heated chamber and images were collected using the IncuCyte^®^ Zoom platform (Essen Bioscience; 20×, NA 0.60, air objective). Phase images were collected every one hour for a total of 12 hours using the IncuCyte^®^ acquisition software. Proteolysis-dependent invasion was calculated by subtracting the relative invasion in the presence of GM6001 from the relative invasion without the GM6001 inhibitor.

### 2D single cell random migration assay

6 well microtiter plates were coated with 10 μg/ml fibronectin and blocked with 1% denatured BSA. Cells were plated at 60,000 cells/well in DMEM/10% FBS containing a final concentration of inhibitors or DMSO as control and allowed to adhere for 12–16 hours. Plates were placed in a 37° C heated chamber and images were collected using the IncuCyte^®^ Zoom platform (Essen Bioscience; 20×, NA 0.60, air objective). Phase images were collected every one hour for a total of 12 hours using the IncuCyte^®^ acquisition software. Trajectory plots, accumulated distance (total cell path length), euclidian distance (the shortest distance between the starting point and end point of migration) and velocity were calculated using the Chemotaxis and Migration Tool (ibidi GMBH).

### 2.5D cell invasion assay

96 well Image Lock microtiter plates (Essen Bioscience) were coated with 100 μg/ml Matrigel (Corning, cat # 354234) and allowed to polymerize overnight at 37° C. MDA-MB-231 cells were plated in a final concentration of 35,000 cells/ml and allowed to adhere for 12–16 hours. A top layer of Matrigel was then added to a final concentration of 2 mg/ml and allowed to polymerize for 30 minutes at 37° C. Inhibitors or DMSO were then added in DMEM/10%FBS to a final concentration of 10 μM with or without MMP inhibitor (GM6001, EMD Millipore; 25 μM final concentration). Plates were placed in a 37° C heated chamber and images were collected using the IncuCyte^®^ Zoom platform (Essen Bioscience; 20×, NA 0.60, air objective). Phase images were collected every one hour for a total of 12 hours using the IncuCyte^®^ acquisition software. Trajectory plots, accumulated distance (total cell path length), euclidian distance (the shortest distance between the starting point and end point of migration), and velocity were calculated using the Chemotaxis and Migration Tool (ibidi GMBH). Proteolysis-dependent invasion was calculated by subtracting the relative invasion in the presence of 25 μM GM6001 from the relative invasion without the GM6001 inhibitor.

### Chemotactic migration/invasion assay

The upper surfaces of 8.0 µm Transwell supports (Greiner Bio-One) were coated with 20µl of growth factor reduced Matrigel (2.5 mg/ml; BD biosciences) for 1 h at 37° C. Excess Matrigel was removed and both chambers were allowed to equilibrate in plain DMEM at 37° C for 1 hour. Following equilibration, the bottom chamber was filled with 750 µl of DMEM/10% FBS containing inhibitors or DMSO control. 50,000 cells were re-suspended in 250 µl of DMEM/0.5% FBS containing inhibitors or DMSO control, plated in the upper chamber, and allowed to invade for 24 hours. Prior to fixation, cells that did not invade were removed from the upper surface of the membranes using a cotton swab. Membranes were fixed in ice-cold methanol for 10 minutes, stained with cell stain solution (Cell Biolabs), washed extensively with DDW, and allowed to dry overnight. Intact membranes were imaged using a Nikon Eclipse TS100 inverted microscope (10×, NA 0.25, air objective). For chemotactic migration assays, cells were plated in the upper chamber of Transwell supports without Matrigel coating and allowed to migrate for 24 hours followed by fixation and staining as above. Invasion index was calculated by subtracting values of chemotactic migration through un-coated membranes from values of chemotactic invasion through Matrigel-coated membranes.

### Mouse xenograft model

All experimental procedures were conducted in accordance with the Federation of Laboratory Animal Science Associations (FELSA) and were approved by the Bar-Ilan University animal care and use committee. Mouse xenograft tumors were generated by injecting a total of 2 × 10^6^ MDA-MB-231/Dendra2 cells re-suspended in 20% collagen I (BD Biosciences) in PBS into the lower left mammary gland of 10-week-old SCID-NOD female mice. Eight weeks later when tumors reached the size of 100 mm^3^, mice were treated by oral gavage with vehicle (5% DMSO, 2% hydroxypropyl methylcellulose, 0.5% Tween-80), imatinib (100 mg/kg), nilotinib (70 mg/kg), or GNF-5 (100 mg/kg) once a day, five days a week, for four weeks. Tumor growth was measured with a caliper twice a week and the tumor volume was calculated by the formula: Tumor volume (mm^3^) = ½ (length × width^2^).

### Tumor immunohistochemistry

SCID-NOD female mice bearing MDA-MB-231/Dendra2 or Arg-YFP, cortactin-TagRFP, MMP-Sense mammary tumors were sacrificed and tumors or lungs were excised, fixed in 4% paraformaldehyde overnight, washed for 1 hour in cold PBS and dehydrated overnight in 30% sucrose. Next, tissue was embedded in OCT and 5 μm thick cryostat sections were placed on silane-coated slides and dried at room temperature followed by permeabilization with 0.1% Triton X-100 for 15 minutes and blocking in 1% BSA and 1% FBS for 1 hour at room temperature. Samples were then incubated overnight at 4° C with the indicated primary antibodies, washed and incubated with the appropriate secondary antibodies. Nuclei were counterstained with 4’,6-diamino-2-phenylindole (DAPI). Tissue was imaged using an inverted laser scanning confocal microscope (Zeiss LSM780; 63×, NA 1.4, oil objective, ZEN black edition acquisition software).

### *In vivo* invasion assay

Cell collection into needles placed into live anesthetized mice was performed as previously described [8]. Briefly, 33-gauge needles were filled with Matrigel and L15-BSA with or without addition of 25 nM human recombinant EGF (Thermo Fisher Scientific). Mice were anesthetized using 5% isoflurane and laid on their back. The isoflurane was reduced to 2%, and a small patch of fur over the tumor was removed. Six 25-gauge guide needles were introduced into the tumor to a depth of 1 mm from both sides. Empty control 33-gauge needles were pushed inside the tumor through the guiding needles to a depth of 2 mm in order to make a path. Control needles were then replaced with Matrigel-filled 33-gauge needles, supplemented with or without EGF. The needles were left in the tumor for 4 hours. Isoflurane concentration was slowly lowered to 0.5% during the course of experiment to keep the mouse breathing even and unlabored. After 4 hours of collection, the needles are removed and the total number of cells collected was determined by 4’,6-diamidino-2-phenylindole (DAPI) staining.

### *In vivo* MMP activation assay

SCID-NOD female mice bearing 1–1.2 cm in diameter MDA-MB-231/Dendra2 mammary tumors were tail vein-injected with MMP Sense 645 FAST (4 nmol in 100 μl of PBS). 24 hours following injection, mice were sacrificed and tumors were excised and processed for immunofluorescence. Cleavage of MMP Sense 645 FAST was analyzed by quantifying the average fluorescent signal area in pixels per field using ImageJ.

### Spontaneous lung metastasis assay

Spontaneous lung metastasis was measured in SCID-NOD mice bearing orthotopic MDA-MB-231/Dendra2-derived tumors of equal size (1–1.2 cm in diameter). Lungs were excised, and the largest lung lobe of each mouse was imaged using a Zeiss Axio observer inverted fluorescence microscope. Single extravascular cells and micro-metastases were counted.

### Distant metastasis free survival analysis

Microarray datasets with Distant Metastasis Free Survival (DMFS) annotation were obtained from the NCBI Gene Expression Omnibus (GEO) data repository [73] of high-throughput microarray experimental data. The meta-cohort dataset comprised of 1,650 tumor expression profiles of primary invasive breast cancer based on the Affymetrix U133 GeneChip microarray platform. Queried transcripts included 22,283 probe sets common to all microarrays in all study populations. Assembly of the datasets was performed using MATLAB (The Mathworks, Inc.) and GEO series (GSE) files were extracted via GEO accessions GSE11121, GSE25055, GSE7390, GSE25065, GSE17705, GSE12093, GSE1456, GSE5327 and GSE45255. The datasets have been retrieved with uniform normalization of probe intensities with MAS 5.0 [74] using global scaling with a trimmed mean target intensity of each array arbitrary set to 600. Cross-population batch effects were corrected using Z-score transformation [75]. The tumor profiles represent primary invasive breast tumors sampled at the time of surgical resection, annotated with DMFS time and censorship status.

Patient samples were split into high and low expressing groups based upon median gene expression. Associations between normalized gene expression and patient survival (DMFS) were assessed by Kaplan–Meier time-event curves and Mantel-Haenszel hazard ratios using an implementation of Kaplan–Meier log rank testing from MATLAB Exchange [76]. All statistical tests were two-sided. The synergy index (SI) was calculated as a means of evaluating additive interaction [77, 78]. The synergy index can be interpreted as the excess risk from overexpression of both genes relative to the risk of overexpression of the genes separately. SI of 1 indicates no synergism, and an SI >1 indicates synergistic interaction between the two genes.

### Overall/disease-free survival and proteogenomic analysis

To analyze overall survival (OS) and disease-free survival (DFS), data from two cohorts of 1,080 and 825 breast invasive carcinoma samples was obtained from The Cancer Genome Atlas (TCGA, https://cancergenome.nih.gov/) [79] and analyzed with cBioPortal tools (http://www.cbioportal.org) [80] using MATLAB. The analyzed datasets contain mRNA-seq expression Z-scores (RNA-Seq V2 RSEM) and Agilent microarray mRNA Z-scores computed as the relative expression of an individual gene and tumor to the expression distribution of all samples that are diploid for the gene. Putative copy number alteration (GISTIC) [81] was collected from both cohorts. GISTIC is an algorithm that attempts to identify significantly altered regions of amplification or deletion and uses discrete copy number calls: homozygous deletion; heterozygous loss; neutral; gain; high amplification. Breast cancer samples are annotated with OS and/or DFS time and censorship status. Samples were split into high and low expressing groups based upon median mRNA expression Z-scores. Associations between Z-scores and patient survival (DFS and OS) were assessed by Kaplan–Meier time-event curves and Mantel-Haenszel hazard ratios using an implementation of Kaplan–Meier log rank testing from MATLAB Exchange [76]. All statistical tests were two-sided. Mass-spectrometry based proteomic characterization of 102 breast cancer tumor samples [53] was obtained from the Clinical Proteomic Tumor Analysis Consortium (CPTAC) Data Portal (https://cptac-data-portal.georgetown.edu) [82] with cBioPortal using MATLAB. For each protein target, Z-scores were determined and Pearson correlation and associated *P*-values were calculated across all samples.

### Statistical analysis

For XTT assay, statistical significance was calculated using Kruskal–Wallis one-way analysis of variance. For 3D scratch wound assays, statistical significance was calculated using ANOVA followed by Tukey post-hoc test in IBM SPSS STATISTICA V21 software. For patient database analysis, statistical significance was calculated using log-rank and Student’s *t*-test implemented in MATLAB, as detailed above. For all other experiments, Student’s *t*-test analysis was performed using GraphPad V5. Values were considered statistically significant if the *P* value was ≤ 0.05.

## SUPPLEMENTARY MATERIALS FIGURES AND VIDEOS











## Abbreviations

MMP: matrix metalloproteinase
HER2: human EGF receptor 2
Arg: Abl-related gene
CML: chronic myeloid leukemia
2D: two-dimensional
3D: three-dimensional
RMSD: root-mean-square deviation
EGF: epidermal growth factor
EGFR: epidermal growth factor receptor
FN: fibronectin
YFP: yellow fluorescent protein
RFP: red fluorescent protein
ECM: extracellular matrix
CNA: copy number alteration
DMFS: distant metastasis free survival
OS: overall survival
DFS: disease-free survival
SI: synergy index
ER: estrogen receptor
PR: progesterone receptor
TN: triple negative
