# Supplementary material for: Targeting invadopodia-mediated breast cancer metastasis by using ABL kinase inhibitors
Source: Oncotarget. 2018 Apr 24;9(31):22158–83. doi: 10.18632/oncotarget.25243 (PMC5955141; doi:10.18632/oncotarget.25243)
Supplement: Supplementary file 1 [file oncotarget-09-22158-s001.pdf]

# Targeting invadopodia-mediated breast cancer metastasis by using ABL kinase inhibitors

## SUPPLEMENTARY MATERIALS

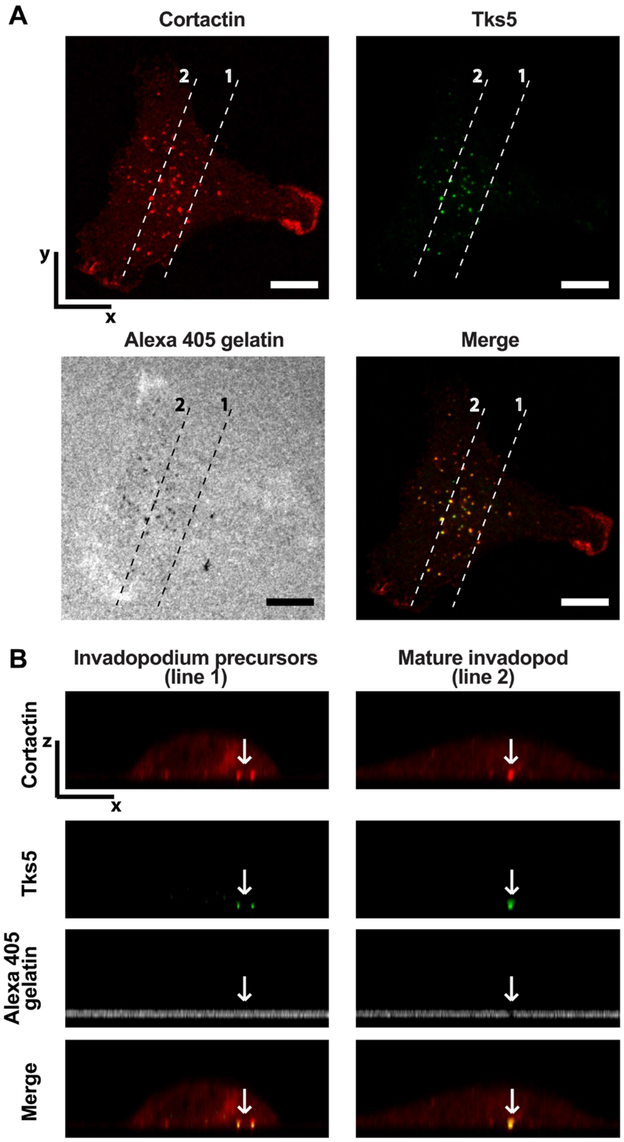


**Supplementary Figure 1: Tks5 and cortactin co-localize at invadopodium precursors and at mature invadopodia.** (**A**) MDA-MB-231 cells were plated on Alexa 405 gelatin, fixed, and labeled for cortactin (red) and Tks5 (green). (**B**) Left: X-Z projection showing co-localization of cortactin and Tks5 at invadopodium precursors (arrow) of a cell plated on Alexa 405 gelatin (white). Right: X-Z projection showing co-localization of cortactin and Tks5 in a mature invadopod penetrating into Alexa 405 gelatin.

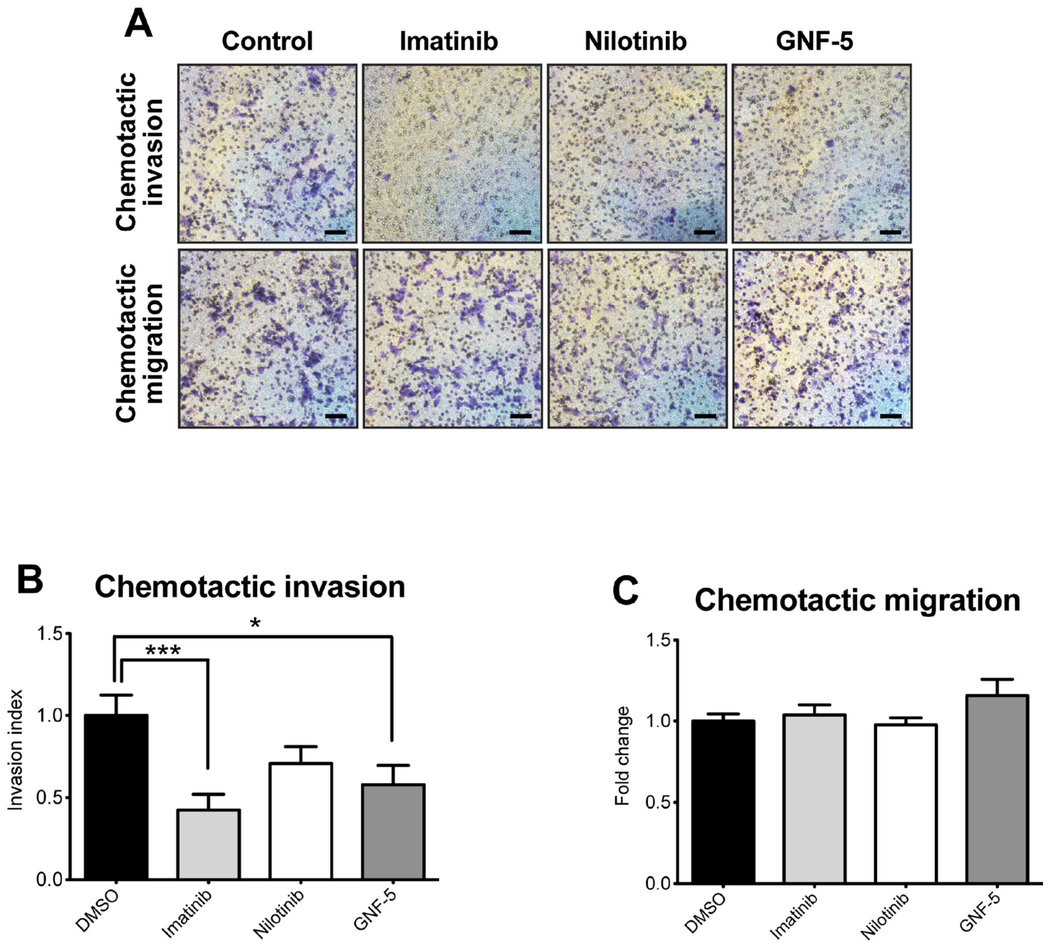


**Supplementary Figure 2: *In vitro* chemotactic invasion, but not migration, is compromised in MDA-MB-231 cells treated with ABL kinase inhibitors.** (**A**) MDA-MB-231 cells were pre-treated overnight with 10 μM imatinib, nilotinib, GNF-5, or DMSO as control and then plated on Matrigel-coated (upper panel) or uncoated (lower panel) membranes, allowed to invade or migrate for 24 hours, fixed and stained. Representative images of cells that invaded or migrated towards the lower part of the membranes are shown. (**B**) Quantification of chemotactic cell invasion through Matrigel-coated membranes. Invasion index was calculated by subtracting the values of chemotactic migration through un-coated membranes from the values of chemotactic invasion through Matrigel-coated membranes. (**C**) Quantification of chemotactic cell migration through un-coated membranes. $n = 28$ (DMSO), $n = 27$ (imatinib), $n = 20$ (nilotinib), $n = 22$ (GNF-5) fields from three independent experiments. $^{*}P \leq 0.05$, $^{***}P \leq 0.001$ as determined by Student's *t* test. Error bars indicate SEM.

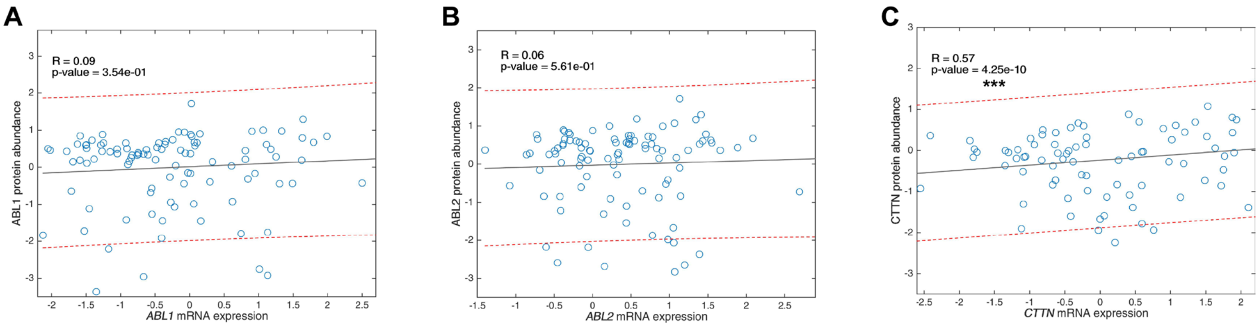


**Supplementary Figure 3: Correlation between Abl, Arg and cortactin mRNA to protein abundance.** Pearson correlation between mRNA and protein expression for Abl (**A**), Arg (**B**), and cortactin (**C**) is indicated. *P* values were calculated using two-tailed Student's *t*-test.

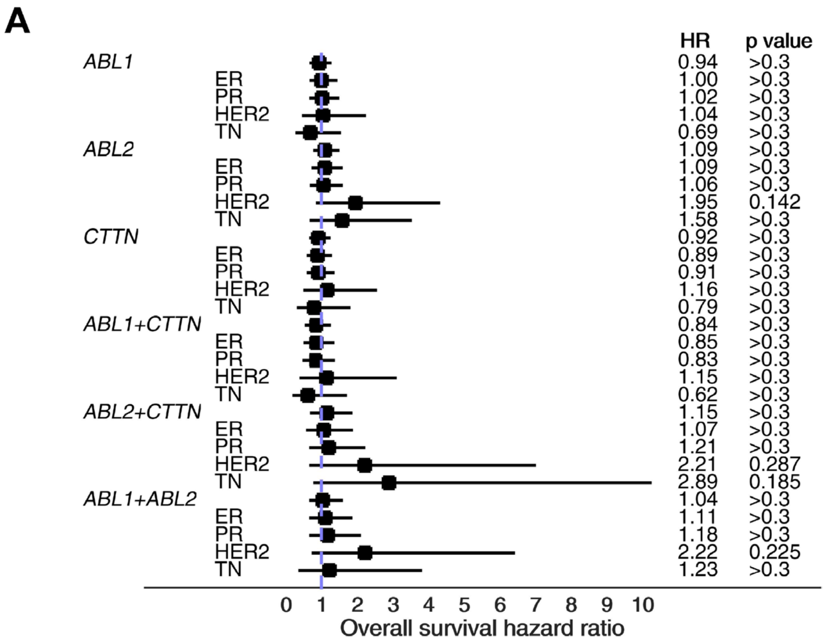


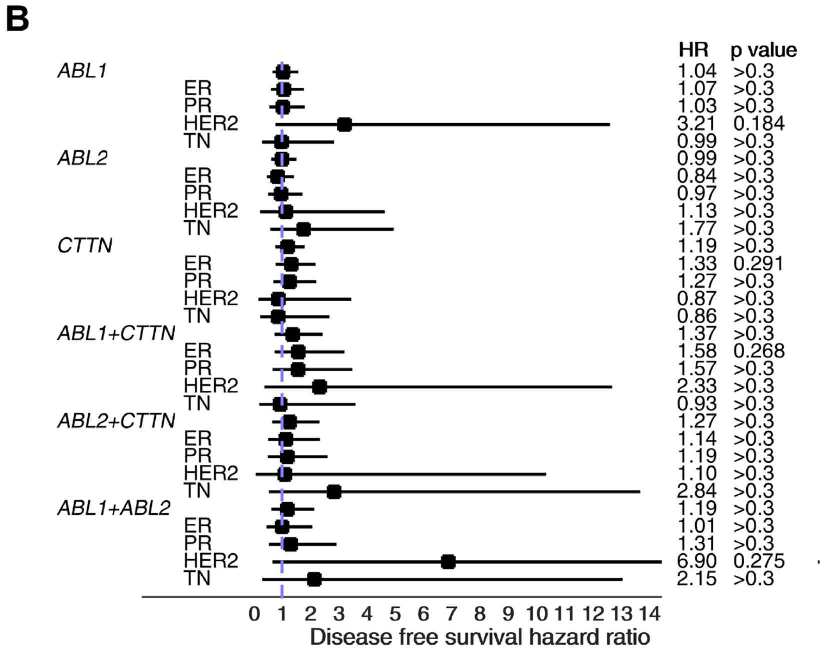


**Supplementary Figure 4: Correlation between *ABL1*, *ABL2*, and *CTTN* mRNA expression and overall survival/ disease-free survival of breast cancer patients.** (**A**, **B**) Overall survival (OS) (A) and disease-free survival (DFS) (B) hazard ratios of ABL1, ABL2, CTTN and their combinations, stratified by hormone receptor status. Tumor samples were split into high and low expressing groups based on mRNA gene expression with Z-score cut-off value 0. Squares indicate hazard ratios and horizontal bars correspond to 95% confidence intervals.

**Supplementary Movie 1: 3D motility of MDA-MB-231 cells treated with DMSO as control.** Representative movie of 3D scratch wound assay of MDA-MB-231 cells embedded in Matrigel and treated with DMSO as control. Movie was acquired every 1 hour for 12 hours. See Supplementary_Movie_1

**Supplementary Movie 2: 3D motility of MDA-MB-231 cells treated with imatinib.** Representative movie of 3D scratch wound assay of MDA-MB-231 cells embedded in Matrigel and treated with imatinib. Movie was acquired every 1 hour for 12 hours. See Supplementary_Movie_2

**Supplementary Movie 3: 3D motility of MDA-MB-231 cells treated with nilotinib.** Representative movie of 3D scratch wound assay of MDA-MB-231 cells embedded in Matrigel and treated with nilotinib. Movie was acquired every 1 hour for 12 hours. See Supplementary_Movie_3

**Supplementary Movie 4: 3D motility of MDA-MB-231 cells treated with GNF-5.** Representative movie of 3D scratch wound assay of MDA-MB-231 cells embedded in Matrigel and treated with GNF-5. Movie was acquired every 1 hour for 12 hours. See Supplementary_Movie_4
